# Asynchronous abundance fluctuations can drive giant genotype frequency fluctuations

**DOI:** 10.1101/2024.02.23.581776

**Published:** 2024-03-23

**Authors:** Joao A Ascensao, Kristen Lok, Oskar Hallatschek

**Affiliations:** 1 Department of Bioengineering, University of California Berkeley, Berkeley, CA, USA; 2 California Institute for Quantitative Biosciences, University of California Berkeley, Berkeley, CA, USA; 3 Present affiliation: Department of Biomedical Engineering, Duke University, Durham, NC, USA; 4 Department of Physics, University of California Berkeley, Berkeley, CA, USA; 5 Department of Integrative Biology, University of California Berkeley, Berkeley, CA, USA; 6 Peter Debye Institute for Soft Matter Physics, Leipzig University, 04103 Leipzig, Germany

## Abstract

Large stochastic population abundance fluctuations are ubiquitous across the tree of life^1–7^, impacting the predictability of population dynamics and influencing eco-evolutionary outcomes. It has generally been thought that these large abundance fluctuations do not strongly impact evolution (in contrast to genetic drift), as the relative frequencies of alleles in the population will be unaffected if the abundance of all alleles fluctuate in unison. However, we argue that large abundance fluctuations can lead to significant genotype frequency fluctuations if different genotypes within a population experience these fluctuations asynchronously. By serially diluting mixtures of two closely related *E. coli* strains, we show that such asynchrony can occur, leading to giant frequency fluctuations that far exceed expectations from models of genetic drift. We develop a flexible, effective model that explains the abundance fluctuations as arising from correlated offspring numbers between individuals, and the large frequency fluctuations result from even slight decoupling in offspring numbers between genotypes. This model accurately describes the observed abundance and frequency fluctuation scaling behaviors. Our findings suggest chaotic dynamics underpin these giant fluctuations, causing initially similar trajectories to diverge exponentially; subtle environmental changes can be magnified, leading to batch correlations in identical growth conditions. Furthermore, we present evidence that such decoupling noise is also present in mixed-genotype *S. cerevisiae* populations. We demonstrate that such decoupling noise can strongly influence evolutionary outcomes, in a manner distinct from genetic drift. Given the generic nature of asynchronous fluctuations, we anticipate they are widespread in biological populations, significantly affecting evolutionary and ecological dynamics.

## Introduction

The dynamics of evolution fundamentally depends on the interplay between the deterministic and stochastic forces acting on populations. Natural selection pushes allele frequencies up or down in the population, depending on the relative allele fitness, while genetic drift causes random allele frequency fluctuations, with no preferred direction^8–12^. Theoretical population genetics has provided many examples of how natural selection and genetic drift can interact with each other. For example, the probability that a mutant will establish in a population is determined primarily by the (stochastic) dynamics dominated by genetic drift at low mutant frequencies, followed by (deterministic) dynamics dominated by natural selection at higher frequencies^10, 13, 14^. The transition point between the two regimes is set by the relative strength of selection and drift. Even in purely neutral scenarios, stochastic forces alone can often lead to surprisingly complex evolutionary dynamics^15–18^.

Ecological dynamics can also be strongly influenced by stochastic demographic fluctuations. It long has been noted that nearly universally, populations across the tree of life exhibit strong abundance fluctuations^6, 19, 20^. In many of the documented cases, the abundance fluctuations follow Taylor’s power law^1–5^ –a power-law relationship between the mean and variance of the abundance fluctuations. Many different ecological processes can cause abundance fluctuations that obey Taylor’s law, including fluctuating environments^21^, spatial effects^1, 22^, or chaotic dynamics^23, 24^. Chaotic population dynamics in particular have captured the interest of ecologists for decades, ever since it was shown that even simple models of single populations can show chaotic dynamics^25^; however, it is generally considered challenging to definitively demonstrate the presence of ecological chaos. Nevertheless, chaotic dynamics have been found in a handful of well-controlled laboratory^26–29^ and field^30–32^ systems, and ecological chaos has recently been suggested to be an underappreciated driver of abundance fluctuations across populations^33^.

Despite the strength and ubiquity of such large population abundance fluctuations, they are not believed to strongly affect the evolutionary dynamics of populations. Evolution is primarily driven by the dynamics of the relative frequency of alleles; if the population is experiencing strong abundance fluctuations, the allele frequencies will be unaffected if all alleles have synchronous abundance fluctuations. Classical genetic drift is a form of demographic stochasticity that arises from independent birth-death randomness, as represented in models such as the Wright-Fisher model^35^. Genetic drift is expected to have a relatively small impact on abundance fluctuations, especially at large population sizes. However, the recognition that ecological dynamics often display giant abundance fluctuations sets the stage for a deeper investigation into their potential evolutionary implications. We propose that giant abundance fluctuations could drive large frequency fluctuations of subpopulations that are unable to synchronize their fluctuations. This hypothesis challenges the traditional models of evolutionary dynamics, suggesting that large abundance fluctuations can sometimes “trickle down” into relative genotype frequencies.

To investigate this, we turned to using genotypes isolated from the *E. coli* Long Term Evolution Experiment (LTEE). The LTEE is a well-known model system in experimental evolution, where several replicate *E. coli* populations have been propagated for over 70,000 generations, evolving in a simple daily dilution environment^36^. The daily dilution environment leads to repeated population bottlenecks, where only one out of every one hundred cells is propagated into the next day’s flask. This bottlenecking is expected to lead to result in genetic drift analogous to that described by the Wright-Fisher model. As a model system, we used two LTEE-derived strains that have coevolved with each other, referred to as *S* and *L*^37–41^. *S* and *L* diverged from each other early in the LTEE evolution, around 6.5k generations, where *S* emerged as an ecologically-distinct, but closely related strain that partially invaded the initially *L*-dominated population^41, 42^.

**Figure 1.**
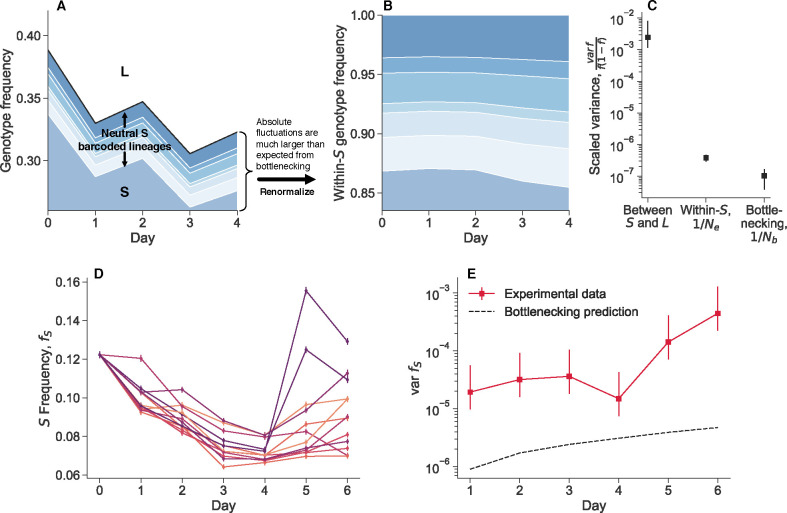
Observation of large genotype frequency fluctuations. **(A, B)** Barcoded libraries of *E. coli* strains *S* and *L* were propagated together in their native serial dilution environment (previously reported data^34^). In a Muller plot representation of lineage sizes, we see that the total frequency of *S* relative to *L* shows large fluctuations. However, neutral barcoded lineages within *S* show substantially smaller fluctuations relative to each other. (**C**) By quantifying the strength of fluctuations, we see that total frequency fluctuations between *S* and *L* are several orders of magnitude larger than fluctuations between neutral lineages and expected fluctuations from bottlenecking. (**D, E**) We propagated replicate cocultures of *S* and *L* strains together, after splitting them from the same mother culture at day zero. Even after one day of propagation, there is already more variance between replicates than expected from bottlenecking, and the variance accumulates over time. Note that the experiments in panels A-C and in D-E were performed at different culture volumes (800mL versus 1mL), but under the same daily dilution rate. All error bars represent 95% CIs.

Ascensao et al. (2023)^34^ previously created random barcoded transposon knockout libraries of *S* and *L*, allowing them to track the frequency dynamics of many subclones of each strain within populations via amplicon sequencing. When they co-cultured the *S* and *L* libraries together, they saw that the total frequency of *S* relative to *L* fluctuated strongly (Figure 1A). In contrast, the fluctuations of neutrally-barcoded variants of *S*, relative to the total *S* population were significantly more muted (Figure 1B). The same holds true for *L* (Figure S1). Quantifying the strength of the observed frequency fluctuations, we see that the fluctuations between *S* and *L* are many orders of magnitude larger than fluctuations within-*S* (Figure 1C). Additionally, the within-*S* fluctuations are similar to the variance expected from bottlenecking, thought to be the primary source of genetic drift in serial transfer environments.

We performed another coculture experiment with *S* and *L*, and measured relative frequencies with flow cytometry, a measurement technique orthogonal to amplicon sequencing. We propagated an *S*/*L* coculture, and then split the coculture into eight replicate cultures at day zero (Figure 1D). We continued to propagate the replicate cultures separately, but in the same environment. After a single growth cycle, there is already more variance between replicates than would be expected from classical genetic drift, and it accumulates over time (Figure 1E). Measurement noise cannot explain the magnitude of the variance, nor the fact that it tends to accumulate over time. We did not find these large fluctuations when we tested another, related pair of diverged genotypes in coculture, REL606 (the LTEE ancestor), and a strongly beneficial mutant, REL606 Δ*pykF* (Figure S2). Instead, we found that the variance accumulation was consistent with classical genetic drift. This indicates that not all non-neutral genotype pairs exhibit these giant fluctuations.

What is the source of these large observed fluctuations, and how do they behave? We find that these giant frequency fluctuations act differently compared to classical genetic drift, leaving a distinctive footprint on the population dynamics. We constructed an effective model that demonstrates how large abundance fluctuations can arise from correlated offspring numbers between individuals. Giant frequency fluctuations originate when the offspring numbers of individuals *within* genotypes are more correlated than those *between* genotypes. We thus refer to such fluctuations as “decoupling noise”. Our analysis further uncovers that these offspring number correlations are primarily driven by underlying chaotic dynamics. These dynamics induce a fluctuating selection-like effect, which significantly influences the population’s evolutionary trajectory. Our findings indicate that decoupling noise is likely common in various biological populations, fundamentally impacting evolutionary and ecological dynamics. This study not only provides a deeper understanding of the mechanisms behind population fluctuations, but also underscores the importance of updating traditional evolutionary theories to integrate these dynamic complications.

## Results

### Empirical fluctuation scaling measurements

We first sought to determine if the large fluctuations we have observed behave in the same way as classical genetic drift. Under neutral drift, the variance in genotype frequency after one generation, varf, will depend on the mean frequency, $var\;f=\langle f\rangle (1-\langle f\rangle )/N_{e}$^35^. At low frequencies, f≪1, the variance will scale linearly with mean, $var\;f\approx \langle f\rangle /N_{e}$. Deviations from the predicted scaling behavior would indicate fluctuations that do not arise from classical genetic drift.

**Figure 2.**
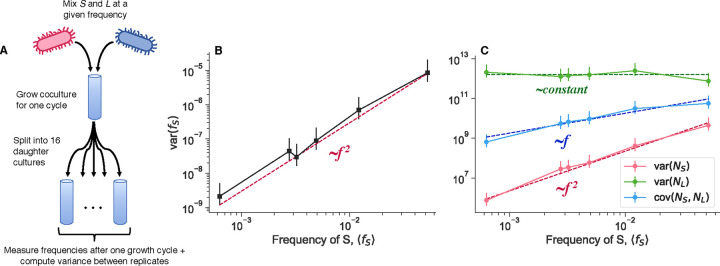
Empirical scaling of population fluctuations. **(A)** After growing *S* and *L* together at defined relative frequencies, we split the cocultures into 16 biological replicates. We then grew the culture for another cycle, and measured the variation across cocultures. We measured **(B)** variance of genotype frequency along with **(C)** variance and covariance of absolute abundance. The points represent experimental measurements, and the dashed lines are fitted lines with the indicated scaling. Error bars represent 95% CIs.

We sought to measure the mean-variance frequency fluctuation scaling relationships in the S and L coculture system, measuring population abundance and frequency via flow cytometry. Briefly, we initially grow each genotype in monoculture for several serial dilution cycles, before mixing the two genotypes together at a defined frequency. After one more growth cycle, we take a flow cytometry measurement of the population, and then split it into biological replicates (Figure 2BC). After another growth cycle, we again take flow cytometry measurements of all biological replicates, and then calculate variances across replicates. We performed the described experiment, varying the initial frequency of the minor genotype, *S*, over about two orders of magnitude, and using 16 biological replicates per initial frequency. The results of the experiment show mean-variance relationships that deviate from the classical genetic drift expectation (Figure S3).

Once we calculate the frequency variance across replicates, we see that the variance scales approximately like $var{\;f}_{S}\propto f_{S}^{2}$ (Figure 2A). We also measured the scaling behavior of the variance of the absolute abundance of S and $L,\;var\left(N_{S}\right)$ and $var\left(N_{L}\right)$ respectively, and the covariance between the two genotypes $cov\left(N_{S},N_{L}\right)$ (Figure 2B). S abundance scales like $var\left(N_{S}\right)\propto f_{S}^{2},\;L$ abundance stays approximately constant, and the covariance scales linearly, $cov\left(N_{S},N_{L}\right)\propto f_{S}$. These observations also deviate from the prediction of classical genetic drift-the variance of S abundance should scale linearly, $var\left(N_{S}\right)\propto f_{S}$, and the covariance should be zero or negative (if the population has a set carrying capacity), $cov\left(N_{S},N_{L}\right)\leq 0$. Furthermore, the data indicate that it is not the case that the fluctuations predominantly arise from only one genotype; S and L abundance fluctuations are of about the same magnitude, with S potentially fluctuating slightly more by a factor of order one (Figure S4). Additionally, we measured total abundance fluctuations as a function of initial population sizes, by varying the volume of the culture while holding the dilution rate constant (Figure S6). We also found that $var(N)\propto N^{2}$, in both monoculture and coculture conditions. This indicates that the power law-scaling abundance fluctuations are present even in the absence of coculture conditions.

Together, these data clearly indicate that the large frequency fluctuations we see cannot be explained by classical genetic drift. Now a new question arises: what type of process is generating the observed fluctuation scaling behaviors?

### Effective model of population fluctuations

We would like to understand how genotype frequency and abundance fluctuations may arise from variation in individuals’ offspring number (SI section S2.1). We first consider a simple population consisting of two genotypes, where all individuals are identical, except for a neutral (genetic) difference to distinguish between the types. There are initially $N_{A}$ and $N_{B}$ individuals of genotype A and B, respectively. Each individual i gives rise to $n_{i}^{\prime }$ net offspring in the next generation, where $\left\langle n_{i}^{\prime }\right\rangle =1$ and $var\left(n_{i}^{\prime }\right)=c_{0}$. We define the total population abundance as $N=N_{A}+N_{B}$, and frequency of genotype A as $f=N_{A}/N$. We can then show that the variance of total population abundance and frequency in the next generation will be,

(1) $$\mathrm{var}N_{\mu }^{\prime }=c_{0}N_{\mu },$$

(2) $$varf^{\prime }\approx c_{0}f(1-f)/N.$$

Thus, we see that we recover the variance relationships for fluctuations due to classical genetic drift, and we explicitly see that classical genetic drift arises from the sum of independent offspring number fluctuations. How can we now extend or generalize this model? One simple extension would be to allow individuals within a population to have correlated offspring numbers. This could arise due to a randomly fluctuating environment which induces transient opportunities (or perils) for reproduction, causing offspring numbers to be coordinated across individuals. We can introduce a new covariance parameter, $cov(n_{i}^{\prime },n_{j}^{\prime })=c_{1}$, for different individuals, i≠j. The variance in population abundance and frequency becomes,

(3) $$\mathrm{var}N_{\mu }^{\prime }=\left(c_{0}-c_{1}\right)N_{\mu }+c_{1}N_{\mu }^{2},$$

(4) $$varf^{\prime }\approx c_{0}f(1-f)/N.$$

**Figure 3.**
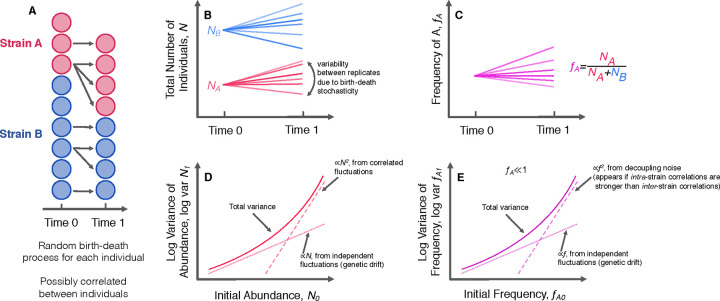
Model of population fluctuations. (**A**) We consider a population of individuals, subdivided into two genotypes. At time zero, there are a specified number of individuals in each genotype. After some period of time, each individual has left behind some random number of descendants, drawn from a distribution that may be correlated between individuals. (**B, C**) The abundance of populations and relative frequencies of genotypes will generally differ across different instances/replicates, due to the random nature of the process. (**D, E**) Our model suggests specific scaling behaviors for both the variance of a total number of individuals in each genotype and variance of the relative frequencies. Specifically, there is a linearly scaling component caused by independent fluctuations (classical genetic drift), and a quadratically scaling component caused by fluctuations correlated between individuals. The quadratically scaling fluctuations will appear in abundance trajectories if there are correlated fluctuations, but they will only appear in frequency trajectories if *intra*-genotype correlations are stronger than *inter*-genotype correlations.

The form of this total population abundance variance scaling has been previously noted^43,44^. The abundance variance now scales linearly at low abundance, but shows quadratic growth at higher abundance. Power law mean-variance scaling of population abundance has been widely observed in ecology, where it is known as Taylor’s power law^1–5^. The variance in frequency stays the same as the case with uncorrelated offspring numbers; intuitively, this is because even though the abundance can strongly fluctuate due to correlated offspring numbers, the population sizes of the two genotypes will fluctuate in sync, so there will be no net effect on frequency fluctuations. However, this will only be the case if the offspring number fluctuations are correlated in precisely the same way with individuals of the same genotype and with individuals of a different genotype. Thus, we will want to consider a yet more general model where the two genotypes are not necessarily identical, and the covariance parameters can depend on the genotypes considered (Figure 3). We consider the offspring number covariance between two individuals, where $n_{\mu ,i}^{\prime }$ represents in the number of net offspring from individual i, which belongs to genotype μ,

(5) $$cov\left(n_{\mu ,i}^{\prime },n_{v,j}^{\prime }\right)=\left\{\begin{array}{ll} c_{0A} & \text{for}\;i=j\;\text{and}\;\mu =v=A\\ c_{0B} & \text{for}\;i=j\;\text{and}\;\mu =v=B\\ c_{1A} & \text{for}\;i\neq j\;\text{and}\;\mu =v=A\\ c_{1B} & \text{for}\;i\neq j\;\text{and}\;\mu =v=B\\ c_{AB} & \text{for}\;i\neq j\;\text{and}\;\mu \neq v \end{array}\right.$$

The abundance variance does not change (equation 3), and the covariance between the abundance of the two genotypes along with the frequency variance becomes,

(6) $$cov\left(N_{A}^{\prime },N_{B}^{\prime }\right)=c_{AB}N_{A}N_{B}=c_{AB}N_{tot}^{2}f_{A}\left(1-f_{A}\right),$$

(7) $$var\left(f_{A}^{\prime }\right)\approx \frac{f_{A}\left(1-f_{A}\right)}{N_{tot}}\left[\left(c_{0A}-c_{1A}\right)\left(1-f_{A}\right)+\left(c_{0B}-c_{1B}\right)f_{A}\right]+\underset{=\delta }{\underbrace{\left(c_{1A}+c_{1B}-2c_{AB}\right.}}f_{A}^{2}{\left(1-f_{A}\right)}^{2}.$$

The new composite parameter δ quantifies the degree of decoupling between the two genotypes. If the genotypes are identical such that $c_{1A}=c_{1B}=c_{AB}$, then the quadratically-scaling fluctuations will vanish. These fluctuations will only appear if the offspring numbers of individuals *within* a genotype are more correlated with each other compared to individuals *between* genotypes. Similar forms for the frequency variance were found by Takahata et al. (1975)^45^ and Melbinger and Vergassola (2015)^21^; however, we consider the more general formulation where all five covariance parameters may differ from each other, and our model can be derived in a more generic way. Our model is readily extensible–we can expand our results to the more general case of a population consisting of m different genotypes (SI section S2.3).

We note that if we consider the case where $c_{0A}-c_{1A}=c_{0B}-c_{1B}$, then we can simplify equation 7,

(8) $$\mathrm{var}\left(f_{A}^{\prime }\right)=\frac{f_{A}\left(1-f_{A}\right)}{N_{e}}+\delta f_{A}^{2}{\left(1-f_{A}\right)}^{2},$$

where the effective population size is defined as $N_{e}=N_{tot}/\left(c_{0A}-c_{1A}\right)$. This form of the variance of genotype frequencies clearly shows that it is composed of two components. The first component arises from independent fluctuations of individuals, linearly scales with frequency, and corresponds to classical genetic drift. The second, quadratically scaling part arises when offspring number fluctuations between genotypes are decoupled to a degree, thus we refer to it as “decoupling noise”. Our model is an effective theory that purports to describe scaling behaviors through covarying offspring numbers; such covariances may arise from a number of different underlying dynamical processes. As previously mentioned, quadratically-scaling abundance fluctuations can arise from fluctuating common environments, which has described as “fluctuating selection”^21,45^. However, fluctuating selection is not the only mechanism that can cause decoupling noise to appear. Various additional mechanisms ^2–5^ can cause the correlated abundance fluctuations that scale like $varN\propto N^{2}$, including inherently chaotic dynamics^23,24^ and spatial effects, such as aggregation and dispersal^1, 22^.

The variance and covariance scaling behaviors in equations 3, 6, 7 are all consistent with the experimentally measured scaling relationships (Figure 2B–C). It appears that the measured range lies in the regime where the linearly-scaling component (classical genetic drift) is negligible compared to the effect of the correlated offspring number fluctuations. There is some evidence that the lowest data point in Figure 2A may fall into the cross-over between the linear and quadratic regimes, but it is not completely clear (Figure S5). The correlation between S and L abundance fluctuations at high frequencies $\left(\rho_{AB}=c_{AB}/\sqrt{c_{1A}c_{1B}}\right)$ is over 90%, demonstrating that a slight decoupling in correlated fluctuations between genotypes is sufficient to generate noticeable decoupling noise. Overall, the quantitative agreement between the experimental data and our model points to the presence of correlated offspring number distributions in the S/L system. However, the origin of such correlated offspring number fluctuations is still not clear.

### Within-cycle growth measurements reveal chaotic dynamics

Populations derived from the *E. coli* LTEE are grown in a serial dilution, glucose minimal media environment, such that the populations are transferred at a 1:100 dilution into fresh media every twenty-four hours. This set-up creates a seasonally-varying environment, where the populations are switching strategies throughout a cycle as it proceeds from feast to famine and back again^39, 46, 47^. We reasoned that the within-cycle dynamics of replicate cultures could help to reveal the origin of decoupling noise. We find evidence that underlying chaotic dynamics are the source of the offspring number correlations between individuals.

We measured the population dynamics of S and L coculture over the course of the twenty-four growth cycle. In a similar protocol as previously described, after several initial monoculture growth cycles, we mixed S and L such that S initially occupies around 6% of the population. After one more growth cycle in coculture, we split the culture into multiple, independent biological replicates, and started to take population measurements over defined time increments via flow cytometry. We grew all of the populations together in a shaking 37°C water bath, to minimize the effects of any possible environmental fluctuations. We measured the dynamics of the first eight hours and those of the last sixteen hours separately (on different days), because we found that the two periods had distinct experimental design requirements. This is primarily because the first eight hours (i.e. during exponential phase) is the period of the fastest dynamics, so we had to use both fewer biological replicates and a more dense sampling strategy.

**Figure 4.**
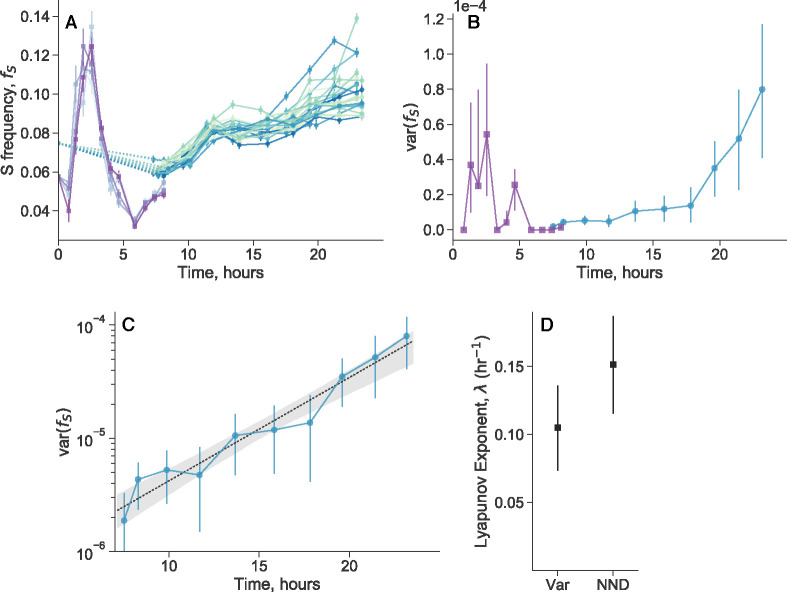
Within-cycle chaotic dynamics of genotype frequencies. (**A**) After splitting cocultures of S and L into multiple biological replicates, we measured genotype frequencies over the course of a twenty-four hour cycle (purple lines: 5 replicates; blue/green lines: 23 replicates). Each line represents a biological replicate. (**B**) Quantifying the variance across replicates over time, we see that the variance peaks both in the first 5 hours, and at the end of the cycle. However, the initial variance is not maintained beyond the first 5 hours, suggesting that the later accumulation of variance is the primary contributor to the decoupling noise. (**C**) We plotted the variance (after 7 hours) on a semilog scale, revealing that the variance appears to increase exponentially over time. The black dashed line represents the exponential fit. Exponentially increasing variance between initially close replicates is indicative of chaotic dynamics. (**D**) Lyapunov exponents calculated from the trajectories after 7 hours, inferred from the exponential fit from the variance (Var) and from the nearest-neighbor distance (NND) method. All error bars represents 95% CIs.

We first look at the within-cycle dynamics of S frequency, $f_{S}$ (Figure 4A). We see relatively complex, out of steady-state dynamics, especially in the first eight hours. As previously described,^48^ the dynamics can be explained by differences in lag time, exponential growth, and stationary phase behavior. The frequency of S initially increases because it “wakes up” from lag phase earlier than L. However, L has a higher growth rate on glucose, so $f_{S}$ starts to decline once L wakes up. Then after a transition period, $f_{S}$ starts to increase again due to a stationary phase advantage and better growth on acetate^39, 48, 49^.

We quantify the variance between replicates, and observe that there are periods of increased variance in approximately the first 5 hours, and the last 7 hours (Figure 4B). The increased variance between replicates in the first 5 hours may be caused by higher measurement error or fast dynamics, but it’s origin is still not clear. The fact that the variance drops close to zero by eight hours, instead of accumulating, suggests a non-biological origin to explain the initial variance. In contrast, after a period where the variance does not increase much, we see a steady accumulation of variance later in the cycle. Specifically, the variance appears to be increasing exponentially at a constant rate, from around 7 hours to the end of the 24 hour cycle (Figure 4C). The data fits an exponential trajectory better than linear or various other non-linear models (Figure S7). Biological replicates continually fluctuate and change their frequency rank order until the end of the time course (Figure S9)–their relative position is not “frozen in” early in the time course.

Exponentially increasing variance between replicates that are initially close to each other is indicative of chaotic dynamics. Chaotic dynamics are classically indicated by extreme sensitivity to tiny perturbations (and a bounded phase space), such that small differences in the initial conditions exponentially increase over time. The observation of exponentially increasing variance is equivalent to the observation of pairwise exponential divergence (SI section S4.1).

We used another standard method to detect chaotic dynamics and infer a largest Lyapunov exponent, based on changes in nearest-neighbor distance (NND)^50^. We inferred a significantly positive Lyapunov exponent (p=0.006), which is consistent with the Lyapunov exponent estimated from the exponentially increasing variance (Figure 4D). The inverse of the Lyapunov exponent (“Lyapunov time”) represents a characteristic timescale of the system, effectively representing how long a system will appear to be predictable. The Lyapunov time is approximately 5–10 hours, implying that trajectories would appear to be stochastic on longer timescales.

Together, these data suggest that decoupling noise originates from underlying chaotic dynamics. Chaotic dynamics cause individuals in a population to coordinate their birth/death rates (in an unpredictable way), creating selection-like effects and leading to mean-variance power law exponents of 2^24^. While various groups have previously seen variations in exponential growth rates^51, 52^ and lag times^53, 54^ across replicates, our data suggest that even if such early-cycle variation exists in our system, it does not significantly impact the final strength of decoupling noise. The overnight timecourses were necessary to reveal the chaotic dynamics, because the system shows a relatively fast Lyapunov time of approximately 5–10 hours, so timepoints taken every 24 hours appear effectively stochastic.

### Extrinsic versus intrinsic decoupling noise

**Figure 5.**
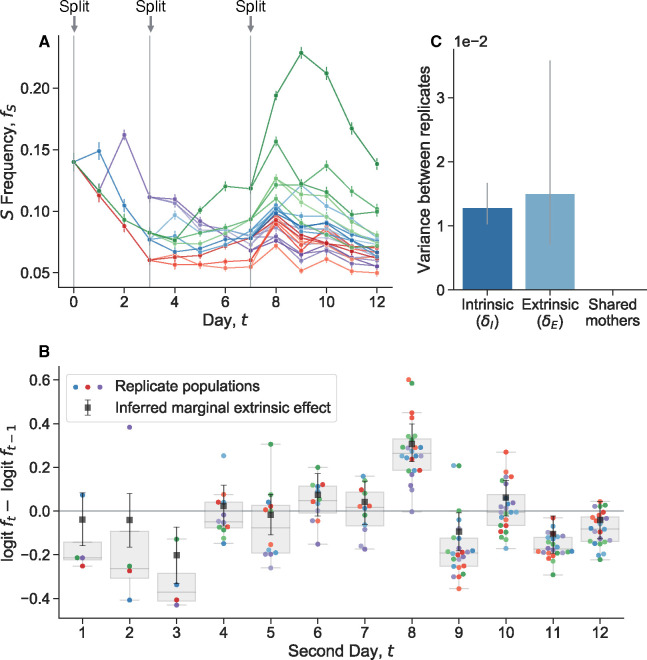
Quantifying the relative strength of intrinsic noise, extrinsic noise, and the effect of sharing mother cultures. (**A**) We performed an experiment where we cocultured S and L together, then split the coculture into four replicate cultures on day 0. We continued to propagate the cultures, and subsequently split each culture into more replicate cultures on days 3 and 7. (B) We computed the change in logit frequency from one day to another (relative, extrinsic fitness effect) for each replicate population. Colors for each population are consistent with panel **A**. The black squares represent the inferred extrinsic “fitness effect” for each day pair, controlling for the effect of frequency-dependent selection, shared mothers, intrinsic noise, and measurement noise. (C) We developed a model to partition variance of frequency noise, reporting the posterior median. All error bars represent 95% CIs.

Most prior experiments that we performed focused on dynamics across one 24 hour growth cycle. However, in both evolution experiments and natural populations, evolutionary and ecological dynamics occurs across many growth cycles or much longer time periods. Our prior experiments were performed over a single growth cycle, with all replicates sharing the same mother culture and experiencing the same environment, controlling for the effects of (potentially subtle) extrinsic environmental noise and between-day memory-like effects. We thus isolated the effects of intrinsic decoupling noise, which we define as the within-day variance component across cultures. Here, we aimed to measure the effects of extrinsic decoupling noise, which we define as the between-day variance component across cultures; this component has also referred to as “fluctuating selection” or “environmental fluctuations”^21,45^. Both intrinsic and extrinsic sources of decoupling noise may contribute to the overall strength of decoupling noise, which could be caused by inherent chaotic dynamics and environmental fluctuations, respectively. We thus conducted an experiment where propagated S/L cocultures over several days in the same environment, serially splitting cultures into new replicates at three different timepoints (Figure 5A).

We see that the frequency of S initially declines across replicate populations, owing to its frequency dependent fitness effect (Figure S10). The populations stabilize around their equilibrium frequency, but continue to fluctuate. We quantified the change in logit frequency from one day to the next for each replicate population (Figure 5B), i.e. the between-day, extrinsic “fitness effect”. We see that there are several days where the change in frequency is noticeably correlated across replicates; for example, from days 7 to 8, most replicates appear to increase in frequency, even though they were at a large range of different frequencies. This seems to indicate that there are significant extrinsic fitness fluctuations, putatively caused by subtle environmental noise. However, there are various possible contributions to fluctuations at each day, including measurement noise, extrinsic and intrinsic decoupling noise, frequency-dependent fitness, and any memory-like effects from sharing a mother culture. To pull apart the contributions of each effect, we built a bayesian hierarchical model and fit it to the data. We see that intrinsic and extrinsic noise contribute roughly the same level of variance to frequency fluctuations (Figure 5C). In contrast, there is little detectable effect of sharing a mother culture, indicating that any memory-like effects are overshadowed by inherent and environmental fluctuations. The large extrinsic fluctuations are perhaps surprising, given that the cultures were maintained in the same temperature and humidity-controlled incubator for the duration of the experiment. Similar sensitivity to putatively subtle environmental fluctuations has been observed in other experiments^55^. This sensitivity is likely caused by the underlying chaotic dynamics of the process, which can exponentially amplify minor environmental differences.

**Figure 6.**
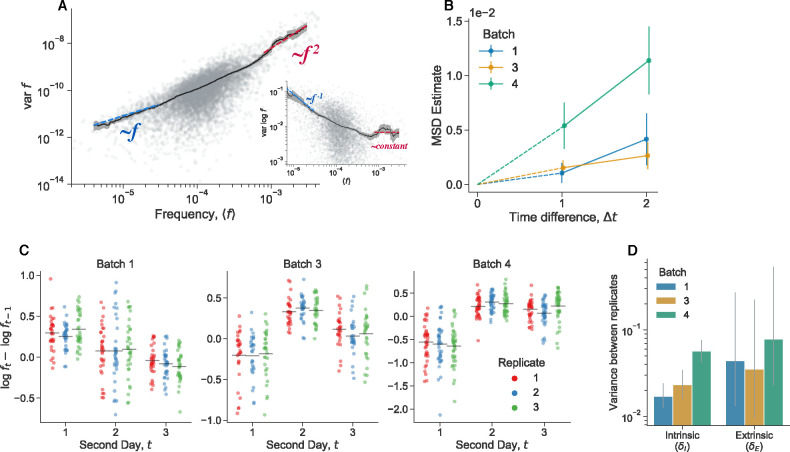
Extrinsic and intrinsic decoupling noise found in barcoded S. cerevisiae populations. Data reanalyzed from Venkataram et al. (2016)^56^. (**A**) We computed the barcode frequency variance between three biological replicates, one growth cycle after the culture was split into the replicates. We included all barcoded mutants in the analysis. Inset shows a different representation of the same data, computing the variance of the log-frequencies. The grey points represent the variance of individal barcoded mutants across the three biological replicates. The black line represents a rolling average. Colored dashed lines represent the indicated scaling. (**B**) Estimate of the mean squared displacement (MSD) of the log-frequencies (restricted to barcodes with high mean frequency). (**C**) Change in log-frequency from one day to another (relative, extrinsic fitness effect) for each barcode in each batch (again, restricted to high frequency barcodes). The gray line represents the mean displacement for each replicate at each timepoint. (**D**) Estimated contribution of both intrinsic and extrinsic decoupling noise to frequency fluctuations. All error bars represent 95% CIs.

Both intrinsic and extrinsic decoupling noise appears to be present in other, unrelated experimental systems. Venkataram et al. (2016)^56^ used a barcoding system to track frequency trajectories of many adaptive variants of *S*. *cerevisiae* yeast. They also find large frequency fluctuations when adaptive variants are at high frequencies. Venkataram et al. cultured populations together, and then split the cultures into three biological replicates at time point 1. We exclude batch 2 from our analysis because it only had two replicate cultures per time point, compared to three replicate cultures for batches 1,3, and 4. We computed the frequency variance between biological replicates after one growth cycle apart (Figure 6A), leveraging the presence of many adaptive barcoded clones to average across the clones to obtain a more precise mean-variance relationship. We pooled all barcoded mutants together in this analysis, and thus we are averaging over the effects across genotypes. We see that the there is an uptick in the variance at high mean frequencies. Specifically, variance in frequency between biological replicates scales approximately like ~f at low frequencies and $\sim f^{2}$ at high frequencies, again consistent with equation 7. An unconstrained power-law fit on the raw data yields a power-law exponent of 1.14 ± 0.05 at low frequencies, and 2.0 ± 0.08 at high frequencies. We also computed the relationship between the mean variance and the variance of log frequencies (Figure 6A inset), which is also consistent with our model, as we would expect a constant mean-variance relationship at high frequencies (the log transformation acts as a variance-stabilizing transform). To investigate if the putative decoupling noise accumulates over time, we estimated the mean-squared displacement (MSD) of the log-frequency trajectories (of barcodes with high mean frequency), correcting for measurement noise and fitness effects (Figure 6B). If decoupling noise is indeed causing the observed quadratic variance scaling at high mean frequencies, the MSD should increase at an approximately linear rate, with the slope corresponding to $\delta_{I}$. Consistently with our prediction, we see that the MSD increases with increasing time increments across all four batches. The strength of intrinsic decoupling noise is approximately the same across the two major classes of adaptive mutants, adaptive haploids and diploids (Figure S13); virtually all high-frequency barcodes are adaptive mutants. If two clones were identical, we would expect that decoupling fluctuations would affect them in the same way, and thus their frequency displacements would be perfectly correlated. Indeed, we see that if two clones are of the same mutant class, their fluctuations are more correlated on average than between clones of different mutant classes (Figure S14).

Similarly to the previously presented data, we investigated the effect of extrinsic noise by plotting the log displacement of high-frequency barcodes over time (Figures 6C). We see that the mean displacement of barcodes is often correlated at time points, in a way that is consistent within batches, but not between batches. This is potentially a signal of extrinsic decoupling noise. To quantify the relative strength of intrinsic versus extrinsic decoupling noise, we employ a similar bayesian hierarchical model to the one previously presented (Figures 6D). We again infer relatively large strengths of both intrinsic and extrinsic decoupling noise. The inferred strength of extrinsic decoupling noise has wide, uncertain posterior across all batches, which is due to the fact that there are a small number of timepoints per batch. However, the error bars provide a lower bound on plausible values of $\delta_{E}$, which is of the same magnitude as the inferred strength of intrinsic noise. Together, these data provide evidence for the presence of both intrinsic and extrinsic decoupling noise in an experimental barcoded yeast system.

The effect of extrinsic fluctuations can easily be incorporated into our model (SI section S2.2). Specifically, if the environment has a negligible autocorrelation time, the total decoupling parameter is simply the sum of the intrinsic and extrinsic components, $\delta =\delta_{I}+\delta_{E}$. Significant environmental autocorrelation times could lead to more complex dynamics^57^. In principle, the extrinsic component can be altered easily by changing the rate/amplitude of environment fluctuations, while the intrinsic component depends on the inherent (chaotic) dynamics of the system.

### Evolutionary implications of decoupling noise

As fluctuations from classical genetic drift have strong effects on evolutionary outcomes, it is reasonable to expect that the presence of decoupling noise may also have evolutionary implications. To study the implications of decoupling noise, we consider our model in the diffusion limit, under constant selection. Previous studies^21, 45, 58^ have studied similar stochastic processes, but we consider the more general form where all five covariance parameters may differ from each other. We obtain analytical results for the fixation probability and the site frequency spectrum, and compare those results to simulations.

#### Fixation probability

**Figure 7.**
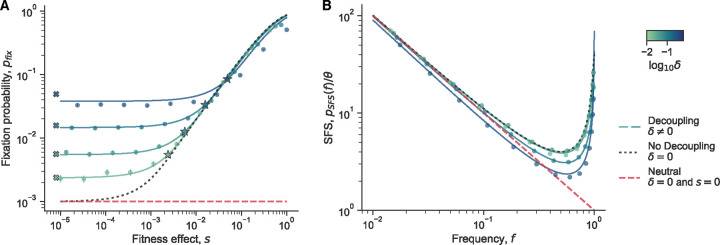
Theoretical evolutionary implications of decoupling noise. **(A)** The fixation probability of a beneficial mutant, as a function of its fitness effect. The star markers show the location of $s_{e}^{*}$ (equation 9), the approximate transition point between the two regimes of $p_{fix}$. The x markers represent the asymptotic fixation probability at low s (equation 10). (**B**) The site frequency spectrum as a function of the mutant frequency. In both plots, the solid lines represent full analytical solutions for dynamics with decoupling noise, across different values of δ. The round markers show simulation results; error bars represents 95% CIs. The black dotted lines represent the case where there are no decoupling noise, but there is still (constant) natural selection. The red dashed lines represents the case where there is neither decoupling noise nor natural selection. Across both plots, $c_{1A}=c_{1B},\;\rho_{AB}=0.5,\;N_{e}=10^{3}$. In (**A**), $f_{0}=10^{-3}$ and in (**B**), s=0.02.

We first consider the fixation probability of a beneficial mutant in a two-genotype system (Figure 7A), i.e. the probability that the mutant completely takes over the population (and that the wild-type goes extinct). We find a general closed form expression for the fixation probability in terms of the initial mutant frequency $f_{0}$ (SI section S3.1).

We first focus on the case where the classical genetic drift parameters are the same between genotypes $\left(\kappa_{A}=\kappa_{B}=1/N_{e}\right.)$, but we can generalize beyond this case (SI section S3.1). The effect of selection is encapsulated by a new effective fitness effect, $s_{e}=s+\left(c_{1B}-c_{1A}\right)/2$. In the limit of weak decoupling noise, $\delta \ll 1/N_{e}$, we find that $p_{fix}$ reduces to the classical fixation probability of a mutant under constant selection^35^, as expected. We then examine the limit where decoupling noise is stronger than classical genetic drift, $\delta \gg 1/N_{e}$, and when the initial frequency is small, $f_{0}\ll 1$. We see that at low $s_{e}$, the fixation probability stays at an approximately constant value (Figure 7A). At higher fitness effects, the fixation probability reduces to the classical expression. The transition point between the two regimes is approximately,

(9) $$s_{e}^{*}\approx \frac{\delta /2+1/N_{e}}{log\left(\delta N_{e}\right)}.$$

As the fixation probability displays two different behaviors in two limits, we can rewrite the fixation probability in an approximate piecewise form,

(10) $$p_{fix}\left(f_{0}\right)\approx \left\{\begin{array}{ll} f_{0}N_{e}s_{e}^{*} & \text{for}\;s_{e}\ll s_{e}^{*}\\ f_{0}\frac{2s_{e}N_{e}}{1-e^{-2s_{e}N_{e}}} & \text{for}\;s_{e}\gg s_{e}^{*} \end{array}\right.$$

The expected transition point and piecewise approximation agree well with both the exact $p_{\text{fix}}$ expression and simulations (Figure 7A). Simulation results start to diverge from the analytical expression at higher δ as the first-order, small frequency deviation assumption starts to break down. Similarly, the small-$s_{e}$ asymptotic prediction also begins to disagree with simulations/analytics at higher δ due to the small $f_{0}$ approximation breaking down, as it is relative to δ (equation S35).

When classical genetic drift is dominant ($\delta \ll 1/N_{e}$) and selection is weak, the fixation probability is simply $f_{0}$. We can thus confirm that the corresponding fixation probability when decoupling noise is dominant $\left(\delta \gg 1/N_{e}\right.)$ differs by a constant factor of $N_{e}s_{e}^{*}$. The transition point $s_{e}^{*}$ acts as a “decoupling-drift barrier”, as selection is unable to efficiently distinguish between genotypes with different fitness effects below this threshold. This threshold can be many orders of magnitude larger than the classical “drift barrier”, which is simple $1/N_{e}$. The transition point also represents an effective boundary, above which the effects of decoupling noise can be safely ignored when computing fixation probabilities.

#### Site frequency spectrum

The site frequency spectrum (SFS) is a commonly used summary of the genetic diversity within a population. It describes the expected density of derived alleles at a given frequency; specifically $p_{SFS}(f)df$ is the number of derived alleles in the frequency range [f-df/2,f+df/2]^59^. Different dynamical processes can leave different characteristic signatures on the site frequency spectrum, so empirical site frequency spectra are often measured to infer aspects of the underlying evolutionary dynamics.

We calculate the SFS for alleles affected by decoupling noise by leveraging previously described approaches^60,61^. We find a general closed form solution for the SFS with constant selection and decoupling noise (SI section S3.2). We see that at low frequencies, the SFS both with and without decoupling noise decays as ~1/f, which is also the expectation for purely neutral alleles^35^ (Figure 7B). Simulations generally agree well with the analytics, but we see again that they start to deviate at higher δ. The major difference between the SFS with and without decoupling noise lies in the uptick at high frequencies, where the uptick rises more quickly when the decoupling mutations are present (Figure 7B). Specifically, in the absence of decoupling noise (and when $\kappa_{A}=\kappa_{B}=1/N_{e}$), the asymptotic scaling behavior of the SFS as $f_{A}\to 1$ is,

(11) $$p_{SFS}\left(f_{A}\right)\sim f_{A}^{N_{e}s-1}$$

In contrast, in the presence of decoupling mutations, we find a different asymptotic scaling behavior,

(12) $$p_{SFS}\left(f_{A}\right)\sim f_{A}^{N_{e}\left(s+c_{1B}-c_{AB}\right)-1}$$

The magnitude of the power-law exponent is not sufficient to distinguish the effects of constant selection and decoupling noise. Thus, decoupling noise can leave selection-like signatures in the SFS, even when the fluctuations do not represent genuine selective forces.

## Discussion

In this study, we show how an ecological mechanism can induce anomalous frequency fluctuations that are much larger than what can be attributed to classical genetic drift or measurement error. While classical genetic drift arises from independent offspring number fluctuations, our analysis suggests that these giant fluctuations arise when the offspring numbers of individuals are more correlated *within* genotypes compared to *between* genotypes. We constructed a simple and generic effective model of genotype frequency fluctuations, revealing that their magnitude is the sum of linearly-scaling classical genetic drift, and quadratically-scaling decoupling noise. By measuring the scaling relationships of the population fluctuations, we showed that the observed large fluctuations were indeed caused by decoupling noise.

Population abundance fluctuations are a well known, near-universal feature of populations across the tree of life, many of which follow Taylor’s power law^1–5^. Our effective model provides a general description of abundance fluctuations with few assumptions, valid for a range of underlying mechanistic processes. Fluctuating selection is one possible mechanism to explain such abundance fluctuations^21,45^, but importantly, other mechanisms such as chaotic dynamics^23,24^ and spatial effects^1,22^ can also cause such fluctuations. Importantly, our model predicts that any population composed of two or more genotypes that have sufficiently decorrelated offspring number fluctuations will experience decoupling noise. Many populations experience abundance fluctuations with a Taylor’s law exponent near 21^9, 20^, including unrelated experimental microbial populations^7^, and thus have correlated offspring number fluctuations. Decoupling frequency fluctuations are thus likely common, especially if offspring number correlations have a genetic basis and are mutable.

In our system, the correlated offspring numbers, and decoupling noise, appear to be caused by underlying chaotic dynamics. The chaotic dynamics can produce a selection-like effect, leading to mean-variance power law exponents of 2^24^. Chaotic dynamics are known to be possible even in simple, single-genotype populations^25, 62^. The fluctuations appear even under the most tightly controlled environment we could create–among replicates from the same mother culture in the same shaking water bath. Thus, unlike fluctuating selection caused by a varying environment, we believe that this decoupling noise is inescapable, and an intrinsic aspect of the system. The underlying source of the decoupling noise in the unrelated barcoded S. cerevisiae populations that we reanalyzed^55, 56, 63^ remains unclear. As previously mentioned, a number of mechanisms are known to cause giant abundance fluctuations^2–5, 21, 23, 24, 45^. However, the recently reported extreme sensitivity of barcoded yeast populations to subtle variations in the environment^55^ is consistent with chaotic dynamics. Chaotic abundance dynamics have been suggested to be common among wild populations^33^, and have been demonstrated in a number of carefully controlled laboratory^26–29^ and field^30–32^ systems. Together, this opens the possibility that chaotic dynamics are also common in experimental microbial populations, and could play an important role in evolutionary dynamics by influencing genotype frequency dynamics across different systems.

The results of our study have implications for the inference of fitness effects in diverse biological populations. Among other possible mechanisms, subtle environmental fluctuations can be amplified by the chaotic dynamics, leading to significant extrinsic decoupling noise, which can cause batch correlations among replicates grown in the same conditions. These batch correlations may be mistaken for a genuine fitness effect, especially if the autocorrelation time of the environment is on a similar timescale as the evolution experiment. Thus, special care must be taken when designing evolution experiments to measure genotype fitness effects. For example, experimenters could perform biological replicates separately on different sets of days, or continuously measure environmental variables (e.g. temperature, humidity) over the time course to quantify the effect of environmental fluctuations. The accuracy of fitness inference procedures may be improved by explicitly modeling the effects of decoupling noise, along with classical genetic drift and measurement noise. Fitness inference methods that leverage temporal correlations between alleles^64^ must be handled with care, lest chaotic or spatial effects be confused with genuine (classical) selection.

Decoupling noise could cause a number of emergent effects on evolutionary dynamics. For example, we showed that the fixation probability of mutants can be drastically shifted by the presence of decoupling noise; thus, the fate of a mutant will not only depend on its fitness effect, but also on its decoupling parameters. The distribution of mutant fitness effects (DFE) is currently thought to largely shape the evolutionary dynamics of adapting populations^65,66^. But the DFE may (partially) lose its predictive power in the face of decoupling noise; we may need to consider a more general joint distribution, between fitness effects, drift effects, and decoupling parameters, especially if the joint distribution has non-trivial structure. For example, if the fitness effects of mutants do not correlate with their decoupling effects, or if decoupling effects are same between mutants, then which mutants become successful would be well predicted by considering the DFE alone. However, if there is a correlation between mutant fitness effects and their decoupling effects, the group of mutants that reach high frequencies and eventually become fixed may differ from what would be anticipated based solely on the DFE. More broadly, the concept of a fitness landscape may need to be updated to a more general fitness-decoupling-drift landscape; evolutionary trajectories through such a landscape will likely differ compared to the case where the decoupling and drift effects are held constant. Significant effort has been devoted to measuring and characterizing the fitness effects of genotypes across systems, i.e. mean offspring numbers. But comparatively little effort has been placed in measuring the drift and decoupling effects of genotypes, i.e. offspring number variance and covariances, even though they can strongly shape evolutionary fates. We thus advocate for an effort to more routinely measure drift and decoupling effects alongside fitness effects.

In our theoretical analysis, we focused on the strong selection-weak mutation regime, where beneficial mutations rise and fix before another establishes. However, it is still unclear how decoupling noise would change the dynamics under other regimes, for example in the clonal interference/multiple mutations regime^67^. Additionally, broad-tail offspring number distributions can emerge out of a diverse array of growth processes^15,68,69^, but their effects on dynamics may change if such distributions are correlated across individuals. Both extensions are likely fruitful avenues for future work.

Overall, we presented experimental measurements for the scaling behavior of population fluctuations, and showed that we could explain them through a generic and extendable theoretical framework. We derived new theoretical results, showing how decoupling noise can impact evolutionary dynamics. We found that decoupling noise, affecting genotype frequencies, can arise quite generally, through a number of mechanisms, so we believe that they may be common across systems.

## Methods

### Growth conditions, media, and strains

All of the experiments presented here were performed in Davis Minimal Media (DM) base [5.36 g/L potassium phosphate (dibasic), 2g/L potassium phosphate (monobasic), 1 g/L ammonium sulfate, 0.5g/L sodium citrate, 0.01% Magnesium sulfate, 0.0002% Thiamine HCl]. The media used in the LTEE and all experiments presented here is DM25, that is DM supplement with 25mg/L glucose. For coculture experiments, we first inoculated the desired strain into 1 mLLB + 0.2% glucose + 20mM pyruvate. After overnight growth, we washed the culture 3 times in DM0 (DM without a carbon source added) by centrifuging it at 2500×*g* for 3 minutes, aspirating the supernatant, and resuspending in DM0. We transferred the washed culture 1:1000 into 1mL DM25 in a glass tube. Generally, we grew 1 mL cultures in a glass 96 well plate (Thomas Scientific 6977B05). We then grew the culture for 24 hours at 37°C in a shaking incubator. The next day, we transferred the cultures 1:100 again into 1 mL DM25. After another 24 hours of growth under the same conditions, we would mix selected cultures at desired frequencies, then transfer the mixture 1:100 to DM25. After another 24 hours of growth under the same conditions, we proceed with the experiment and start collecting measurements.

We used strains with fluorescent proteins inserted at the *att Tn*7 locus, integrated via a miniTn7 transposon system, as previously reported^48^. The 6.5k *S* strain was tagged with eBFP2, the 6.5k *L* strain was tagged with sYFP2, REL606 was tagged with sYFP2, and the REL606 △pykF mutant^70^ was tagged with mScarlet-I.

### Flow cytometry

For all population measurements taken with flow cytometry, we used the ThermoFisher Attune Flow Cytometer (2017 model) at the UC Berkeley QB3 Cell and Tissue Analysis Facility (CTAF). For every measurement, we loaded the samples into a round bottom 96 well plate, for use with the autosampler. We set the flow cytometer to perform one washing and mixing cycle before each measurement, and ran 50μL of bleach through the autosampler in between each measurement to ensure that there was no cross-contamination between wells. We used the “VL1” channel to detect eBFP2 fluorescence, which uses a 405nm laser and a 440/50nm bandpass emission filter. We used the “BL1” channel to detect SYFP2 fluorescence, which uses a 488nm laser and a 530/30nm bandpass emission filter. We used the “YL2” channel to detect mScarlet-I fluorescence, which uses a 561nm laser and a 620/15nm bandpass emission filter. We used a previously described and validated analysis framework^48^ to extract cell counts and strain frequencies from raw flow cytometry data.

### Multi-day timecourses

We analyzed barcode sequencing data previously reported in Ascensao et al. (2023)^34^, focusing on experiment “Eco Eq 1”. We used the $N_{e}$ estimate reported in Ascensao et al. (2023), which was obtained by decomposing within-ecotype neutral barcode frequency fluctuations into a component that accumulates over time (demographic fluctuations), and a component that is uncorrelated over time (measurement noise). We used previously reported data on colony forming units (CFUs) to estimate the bottleneck size, $N_{b}$, by taking the average of barcoded (kanamycin resistant) CFUs over the timecourse. We obtained an estimate of the frequency variance between-ecotypes by fitting a linear model to the time course via ordinary least squares, and computing the variance around the line. We obtain quantitatively consistent results by leveraging the two biological replicates of the experiment, which were split at day 0, by calculating the variance between frequencies at day 1; this estimation method results in much wider confidence intervals, but a lower bound that is still several orders of magnitude larger than expected variance from bottlenecking and classical genetic drift. Similarly large between-ecotype fluctuations were found for all other coculture experiments presented in Ascensao et al. (2023).

We propagated cocultures of S and L (Figure 1D–E), along with cocultures of REL606 and the REL606 △pykF mutant (Figure S2), where we started the cocultures as described above. We split the cocultures into eight replicates at day 0, where all replicates where grown in the same 37°C incubator, at the same time. We took flow cytometry measurements of the populations at the end of each 24 hour cycle. We computed a robust estimate of the variance (to decrease the influence of outliers) through the median absolute deviation, $varf\approx 2.1981\cdot med{\left|f_{i}-{med}_{i}f_{i}\right|}^{2}$. Confidence intervals were determined by standard bootstrapping. To compute the “genetic drift prediction” of how frequency variance should change over time, we used $N_{e}=10^{5}$, which is the approximate (conservative) bottleneck population size for both cocultures. We computed the approximate expected variance due to drift as var $f_{1}=f_{0}\left(1-f_{0}\right)/N_{e}$ for the first time point, and then var $f_{t+1}=varf_{t}+\left\langle f_{t}\right\rangle \left(1-\left\langle f_{t}\right\rangle \right)/N_{e}$ for all subsequent time points.

To obtain estimates of the scaling behavior of variance quantities with respect to the frequency of the minor genotype, S (Figure 2), we first set up S/L cocultures as described above, by mixing the cultures at different frequencies over about two orders of magnitude with S in the minority. We split each coculture into 16 replicate cultures, all grown under the same conditions. After one 24 hour growth cycle, we took three, independent flow cytometry measurements of each culture, which we treated as technical replicates for each culture. We utilized technical replicates for each culture primarily to decrease the effective amount of measurement noise for abundance–abundance measurements are noisier in our system than frequency measurements^48^. We took the average across technical replicates as the final frequency and abundance estimates for each biological replicate culture. We then computed variance across biological replicates with the standard estimator. Quantitatively and qualitatively similar results are obtained by using the robust estimator for the variance. We compared the variance to the mean frequency, instead of the frequency at the beginning of the cycle, because the within-cycle frequency dynamics show that the mean frequency is a better measure of the frequency right before variance starts to accumulate (Figure 4A). We inferred the power-law exponents by performing ordinary least squares regression on the log-transformed variance/covariance quantity against the log-transformed mean S frequency; we determined confidence intervals via standard bootstrapping (Figure S3).

We measured the relationship between initial abundance and variance of abundance (Figure S6) by growing cultures as previously described, mixing S and L at around $f_{S}\approx 0.05$ (the approximate equilibrium frequency) for the coculture condition, as well as continuing to propagate monocultures of S and L. We split the cultures into 16 replicate cultures per volume condition (using the same 1:100 daily dilution rate for all conditions). We used 0.1, 1, and 10mL culture volumes, where used the glass 96 well plates for the first two conditions, and 50 mL glass erlenmeyer flasks for the 10 mL condition. After the 24 hour growth cycle, we plated each replicate culture on LB plates, at a 10^−5^ ML^−1^ dilution rate. We additionally took flow cytometry measurements for each replicate in the coculture condition, to more accurately measure genotype frequency. Confidence intervals were computed by standard bootstrapping. There are no statistically significant differences in variance scaling when comparing the monoculture and coculture conditions, or between S amd L.

### Within-cycle timecourses

After the initial growth cycles of fluorescently tagged S and L as previously described, we mixed the strains together such that the relative frequency of S was around 6%. We grew the coculture for one more cycle in DM25, then took a flow cytometry measurement at the end of the 24 hour cycle, which we took as time 0. We then immediately inoculated new replicate cultures from the overnight mother culture by diluting the culture 1:100 into DM25 (e.g. 300 μL of culture +30 mL of DM25), vortexing the mixture well, and then splitting the resulting mixture into 1 mL cultures in individual wells of a glass 96 well plate. We used 5 biological replicates for the 8 hour time-course and 23 replicates for the 24 hour time-course. We secured the 96 well plate in a 37°C water bath, shaking at 180rpm. The wells of the glass 96 well plate are separated such that water can pass in between the wells. We briefly removed the plate at designated time intervals (about every 30 minutes and 2 hours for the 8 and 24 hour time course, respectively) to subsample approximately 60μL of culture for flow cytometry measurements. Subsamples were discarded after measurement. Exact times of plate sampling were documented. We subsampled the cultures from the 24 hour time course in two batches (one of 11, one of 12), where we subsampled the second batch immediately after the flow cytometry measurement of the first batch was finished. We utilized the batch structure to minimize the amount of time the samples have to wait outside of the water bath to be measured by the flow cytometer.

Following data processing, we computed the variance between biological replicates at the same time points. For the 24 hour time-course, we first computed the variance among all samples in the same batch for each time point, then averaged the variance between the two batches at the same time point, because the batches were taken at slightly different actual times. The confidence intervals for variance measurements were computed with standard asymptotic formulas. We fit various curves (Figure S7A) to the variance trajectory after 7 hours by first log-transforming the variances (as an approximate variance-stabilizing transform), and then performing least squares regression. Denoting $v_{i}$ as the variance and $t_{i}$ as the time, we fit a simple exponential curve, $log\;v_{i}=log\;a+bt_{i}+\varepsilon_{i}$, a linear curve, $log\;v_{i}=log\left(a+bt_{i}\right)+\varepsilon_{i}$, a generalized power-law curve, $log\;v_{i}=log\left(c+at_{i}^{b}\right)+\varepsilon_{i}$, and a quadratic curve, $log\;v_{i}=log\left(c+at_{i}+bt_{i}^{2}\right)+\varepsilon_{i}$. Confidence intervals for the fits were obtained by resampling trajectories of biological replicates with replacement (standard bootstrapping), computing the variance between replicates in the same manner as previously described, and performing the appropriate regression again.

We evaluated the fits of all regressions by computing their Akaike Information Criterion (AIC),

(13) $$\text{AIC}=n\log\left(\frac{1}{n}\sum_{i}{\left({\hat{v}}_{i}-v_{i}\right)}^{2}\right)+2p.$$

Where ${\overset{ˆ}{v}}_{i}$ is the variance predicted from the regression model, n is the number of time points, and p is the number of parameters fit in the model. We observed that the AIC of the exponential fit was lower than all others (Figure S7B). Thus, we sought to determine if this difference was significant. To this end, we computed paired one-sided p-values similarly to how we computed confidence intervals, i.e. with standard bootstapping, re-performing the regression, and calculating the AIC.

We calculated Lyapunov exponents (λ) from the frequency trajectories after seven hours in two ways. First, we implemented and appropriately modified the method proposed by Rosenstein et al. (1993)^50^, based on nearest-neighbor distance (NND) trajectories. The method relies on (1) appropriately embedding the data, (2) identifying the nearest neighbor j at the initial timepoint of each trajectory j (calculated using euclidean distance), (3) computing the euclidean distance between initially nearest neighbors over time, $d_{i,j}(t)$, and (4) fitting an exponential to all computed pairwise distances via least squares regression,

(14) $$\log d_{i,j}\left(t\right)=\lambda t+b+\varepsilon_{i,j}\left(t\right).$$

To embed the data, we first needed to choose the appropriate hyperparameters for the embeddding dimension and lag time, which we did via shuffle splitting cross-validation. Specifically, for each embedding, we shuffled the data with 1000 replicates, then used 30 of the distance points to fit the model shown in equation 14 and obtain and estimate for λ and b. Using the fitted parameters, we computed the mean squared error (MSE) for all remaining out-of-sample points, and averaged the MSE across all shuffled replicates. The one-dimensional case had the lowest out-of-sample MSE, so we proceeded with the one-dimensional Lyapunov exponent estimate (Figure S8B). The Lyapunov exponents obtained for the other hyperparameter sets were very similar to the one-dimensional case (Figure S8A), showcasing the robustness of the method.

Recent work has suggested the use of Lyapunov exponent inference methods based on dynamics reconstruction and jacobian estimation^33^. However, our timecourse is not long enough to accurately reconstruct dynamics in the necessary manner, and would not allow for effective jacobian estimation. Additionally, measurement noise is small in our experimental set-up, and we can leverage the numerous biological replicates starting from (nearly) the same initial conditions to directly look at divergence of trajectories. Thus, we do not believe the use of a jacobian-based inference method is necessary or appropriate for this experiment.

The exponentially increasing variance between biological replicates can also be used to extract a Lyapunov exponent (SI section S4.1). Specifically, var $f\propto e^{2\lambda t}$, so we took the Lyapunov exponent as half of the fitted exponent. This is valid if the system is well-described as one-dimensional (although this assumption could be relaxed in principle); our previous hyperparameter analysis revealed that this was indeed the case. In both methods, we obtained confidence intervals by standard bootstrapping.

### Extrinsic fluctuations and splitting cultures

We started a coculture of S and L with the same protocol as previously described. At day 0, we split the culture into 4 replicate cultures by diluting the culture 1:100 into DM25, vortexing the mixture well, and then splitting the resulting mixture into 1mL cultures in individual wells of a glass 96 well plate. We split replicate cultures at days 3 and 7, via the same procedure, into 3 and 2 new subreplicates for each culture respectively. We took flow cytometry measurements at the end of each growth cycle for twelve days.

We sought to model the effects of measurement noise, frequency-dependent fitness effects, intrinsic and extrinsic decoupling noise, and memory-like effects from sharing mother cultures (from splitting cultures). We used a bayesian hierarchical modeling approach to model the data, as it allowed us to flexibly set up the model, and obtain full posterior estimates. We focused on modeling the logit frequency displacements for each culture, because a logit transform serves as a variance-stabilizing transform for decoupling noise and fitness effects. For example, in the simplest case, considering just fitness effects and intrinsic decoupling noise, the distribution of frequencies at time t is,

(15) $$f_{t+1}|f_{t}\sim N(f_{t}+s\left(f_{t}\right)f_{t}\left(1-f_{t}\right),\delta_{I}f_{t}^{2}{\left(1-f_{t}\right)}^{2}).$$

After a logit transformation, the frequency displacement is,

(16) $$\Delta logit\;f_{t}=logit\;f_{t+1}-logit\;f_{t}\sim N\left(s\left(f_{t}\right),\delta_{I}\right).$$

We model the effect of environmental fluctuations by considering an environmental effect that is drawn from a centered normal distribution at each time point,

(17) $$E_{t}\sim N\left(0,\delta_{E}\right).$$

Similarly, we model the effect of shared mothers as another centered normal distribution,

(18) $$M_{g,t}\sim N\left(0,v_{M}\right).$$

The g subscript indexes a group that all arise the same mother culture at time t, such that all daughter cultures will share the same $M_{g,t}$. We focus on modeling the effect of sharing a mother culture immediately after splitting cultures on days 3 and 7; for all other days, we set $M_{g,t}=0$. Both the effect of shared mothers and environmental fluctuations feed into the mean of the frequency displacement for each timepoint. We model the frequency dependent fitness as a linear function, $\alpha +\beta f_{t,g,i}$, which appears to capture this dependence well in the frequency regime our data lies in (Figure S10). The final hierarchical model that we fit to the data reads as,

(19) $$\Delta logit\;f_{t,g,i}\sim N\left(\alpha +\beta f_{t,g,i}+E_{t}+M_{t,g},\delta_{I}+\mu_{t+1,t,i}\right).$$

We account for measurement noise by specifying $\mu_{t+1,t,i}$; we previously found that frequency measurements have errors well approximated by a binomial distribution in our flow cytometry protocol and set-up^48^. For the logit-transformed frequency displacements, we use a first order approximation for the binomial variance such that,

(20) $$\mu_{t+1,t,i}={\left[f_{t+1,g,i}\left(1-f_{t+1,g,i}\right)n_{t+1,g,i}\right]}^{-1}+{\left[f_{t,g,i}\left(1-f_{t,g,i}\right)n_{t,g,i}\right]}^{-1},$$

where $n_{t,g,i}$ is the total number of cells detected in the flow cytometer.

We fit the model to all of the data (frequency displacements), jointly inferring $\alpha ,\;\beta ,\;\delta_{I},\;E_{t},\;M_{t,g},\;\delta_{E}$, and $v_{M}$. We use the hamiltonian monte carlo (HMC) algorithm implemented in STAN^71^ to jointly estimate the posterior of each parameter. We used a non-centered parameterization of the model to improve convergence of the hierarchical model. For the variance parameters, i.e. $\delta_{E},\;\delta_{I}$, and $v_{M}$, we use the Jeffrey’s prior for variance, $p(x)\propto x^{-2}$. To account for the non-centered parameterization for $E_{t}$ and $M_{t,g}$, we use a standard normal prior, x~N(0,1), then multiplied both by the standard deviation parameters, $E_{t}\to E_{t}\sqrt{\delta_{E}}$ and $M_{t,g}\to M_{t,g}\sqrt{v_{M}}$. For all remaining parameters, we used a uniform prior.

### Reanalysis of barcoded yeast data

We reanalyzed the barcoded yeast strain time-courses from Venkataram et al. (2016)^56^. We excluded batch 2 from our analyses because it only had two biological replicates, while batches 1, 3, and 4 all had three biological replicates each. The serial dilution evolution experiments in Venkataram et al. were conducted such that there was one culture at time point 1, which was sampled for barcode sequencing, which was then split into three (or two) biological replicates, which were all sampled for all subsequent time points. We first computed the mean and variance of each barcode frequency across the three biological replicates at time point 2 (after one growth cycle apart). We only pooled mean-variance data points from batches 1 and 3 in Figure 6A because batch 4 behaved slightly differently than the other two, in that it had stronger decoupling noise (Figure 6B,D). However, the data from batch 4 still has the same asymptotic scaling behaviors (Figure S11). After plotting the relationship between the mean and variance of barcode frequencies, we computed a moving average of the relationship. We computed the moving average of var(f) as a function of ⟨f⟩ with a multiplicative window (i.e. a constant sized window on log⟨f⟩). We went along the x-axis, and computed the average of var(f) in between [⟨f⟩⋅(1-w),⟨f⟩⋅(1+w)], where we set w=0.3. Changing the smoothing parameter w does not significantly alter the results. We computed confidence intervals by standard bootstrapping. We estimated power law exponents by performing ordinary least squares regression on the log-transformed variance against the log-transformed mean. For the low-frequency power-law fit, we used all points below $\langle f\rangle <5\cdot 10^{-5}$. For the high-frequency power-law fit, we used all points above $\langle f\rangle >3\cdot 10^{-4}$. We computed standard errors on the power-law exponents by standard bootstrapping.

We then sought to estimate the mean squared displacement (MSD) of the log-transformed frequencies, at high frequencies such that decoupling noise is dominant (Figure 6B). The log transformation acts a variance-stabilizing transform; we can use this approximation (instead of a logit transform) as all barcodes here are at low frequency f≪1. We expect that the MSD should linearly increase over time increments, with a slope determined by δ,

(21) $$MSD(\Delta t)=\left\langle {\left(log\;{\tilde{f}}_{t}-log\;{\tilde{f}}_{t-\Delta t}\right)}^{2}\right\rangle$$

(22) =δΔt

We estimated the MSD for all pairs of time points that were either 1 or 2 time increments (Δt) apart. We filtered for high frequency barcodes at a mean frequency range from 5 · 10^−4^ to 5 · 10^−3^. We took ${\tilde{f}}_{t}$ as centered barcode frequencies, where we subtracted the average barcode frequency over all replicates in a batch, ${\tilde{f}}_{t}=f_{t}-\left\langle f_{t}\right\rangle$. This is equivalent to subtracting the mean “fitness effect” over a pair of time points. We need a way to separate measurement error (uncorrelated in time) from biological decoupling noise (accumulates over time). We leveraged a previously utilized^34^ method to pull apart the two sources of noise Briefly, with measurement noise, variance between two time points will be the sum of the decoupling noise and measurement noise $\left(\zeta_{t}\right)$ for the two time points,

(23) $$\kappa_{t,t-\Delta t}=\delta \Delta t+\zeta_{t}+\zeta_{t-\Delta t}.$$

For every barcode, we compute the log difference at considered time point pairs, $\phi (t,t-\Delta t)=log\;{\tilde{f}}_{t}-log\;{\tilde{f}}_{t-\Delta t}$. We then obtain a robust estimate of κ through the median absolute deviation, over all barcodes,

$${\overset{ˆ}{\kappa }}_{t,t-\Delta t}\approx 2.1981\cdot med\;|\phi (t,t-\Delta t)-med\;\phi (t,t-\Delta t){|}^{2}.$$

We obtain estimates for the standard error, std ${\overset{ˆ}{\kappa }}_{t,t-\Delta t}$, through standard bootstrapping. With the relationship between $\kappa_{t,t-\Delta t}$ and the noise parameters (equation 23), we can estimate the noise parameters given all of our measured ${\overset{ˆ}{\kappa }}_{t,t-\Delta t}$. We do this by numerically minimizing the weighted squared difference, $\sum {\left[\left(\kappa_{t,t-\Delta t}-{\overset{ˆ}{\kappa }}_{t,t-\Delta t}\right)/std\;{\overset{ˆ}{\kappa }}_{t,t-\Delta t}\right]}^{2}$. We obtain our final estimate of the MSD by subtracting the measurement noise parameters from each ${\overset{ˆ}{\kappa }}_{t,t-\Delta t}$, and averaging over all values with the same time increment Δt,

(25) $$\hat{MSD}\left(\Delta t\right)={\left\langle {\overset{ˆ}{\kappa }}_{t,t-\Delta t}-{\overset{ˆ}{\zeta }}_{t}-{\overset{ˆ}{\zeta }}_{t-\Delta t}\right\rangle }_{\Delta t}.$$

We obtained confidence intervals for the MSD via standard bootstrapping. We are uncertain about the scaling behavior of the measurement noise parameters, relative to the mean frequency. We treat the $\zeta_{t}$ parameters as constant, which should be a valid assumption if the measurement noise does not significantly change over the frequency range that we used. Using an even smaller frequency range does not seem to change our results significantly–we obtain similar results with a thinner mean frequency range of 7 · 10^−4^ to 2 · 10^−3^, albeit with higher error (as expected) (Figure S12).

To model the contributions of intrinsic/extrinsic decoupling noise to the frequency trajectories of high-frequency barcodes, we turned to a similar set-up as previously considered (equation 19). We change the model to reflect the data structure,

(26) $$\Delta \log f_{t,i}\sim N\left(\alpha_{i}+E_{t},\delta_{I}+{\overset{ˆ}{\zeta }}_{t+1}+{\overset{ˆ}{\zeta }}_{t}\right).$$

We eliminated the “sharing mothers” parameters, and we included a barcoded-dependent mean fitness effect $\alpha_{i}$, which we fit for each barcode. We fit the model for each batch in the same manner as previously described. available under aCC-BY 4.0 International license.

## Evolutionary dynamics simulations

We simulated the evolutionary dynamics of a two-genotype system to compare with our theoretical results on the evolutionary implications of decoupling noise, as shown in Figure 7. We directly simulated the number dynamics of each genotype, $N_{i}$, with the Langevin equation (following the approach of Melbinger and Vergassola (2015)^21^),

(27) $$\frac{dN_{i}}{dt}=\left(\mu_{i}-\frac{N_{tot}}{K}\right)N_{i}+N_{i}\xi_{i}\left(t\right)+\sqrt{N_{i}\left(\mu_{i}+\frac{N_{tot}}{K}\right)}\eta_{i}\left(t\right).$$

Here, $N_{tot}=\sum_{i}\;N_{i},\;\eta_{i}(t)$ is standard gaussian white noise, and $\xi_{i}(t)$ is correlated gaussian white noise, such that,

(28) $$\left\langle \xi_{i}(t)\xi_{j}\left(t^{\prime }\right)\right\rangle =\left\{\begin{array}{ll} \delta \left(t-t^{\prime }\right)c_{1A} & \text{for}\;i=j=A\\ \delta \left(t-t^{\prime }\right)c_{1B} & \text{for}\;i=j=B\\ \delta \left(t-t^{\prime }\right)\rho_{AB}\sqrt{c_{1A}c_{1B}} & \text{for}\;i\neq j \end{array}\right.$$

The frequency dynamics, $f=N_{A}/\left(N_{A}+N_{B}\right)$, have previously been approximately derived from the abundance dynamics^21^, using a first order approximation. We see that those frequency dynamics are equivalent to the dynamics we derive (equation S18) when the classical genetic drift parameters are the same between genotypes, $\kappa_{A}=\kappa_{B}$. The fitness effect becomes $s=\mu_{1}-\mu_{2}$, and the effective population size becomes $N_{e}=K/2$. We directly simulated the abundance dynamics in equation 27 by discretizing time,

(29) $$\Delta N_{i}\approx \left(\mu_{i}-\frac{N_{tot}}{K}\right)N_{i}\Delta t+N_{i}{\tilde{\xi }}_{i}\left(t\right)\sqrt{\Delta t}+\sqrt{N_{i}\left(\mu_{i}+\frac{N_{tot}}{K}\right)}{\tilde{\eta }}_{i}\left(t\right)\sqrt{\Delta t}.$$

Now ${\tilde{\eta }}_{i}(t)$ is a standard gaussian random variable, and ${\tilde{\xi }}_{i}(t)$ is a gaussian random variable with mean of 0 and a covariance matrix given by equation 28. We used initial conditions of $N_{A}=10^{-3}K$ and $N_{B}=K$. In our simulations, we consistently used $K/2=10^{3},\;\mu_{A}=1,\;\rho_{AB}=0.5$, and $c_{1}=c_{1A}=c_{1B}$. We set Δt=0.1, which seemed to sufficiently mitigate time-discretization error. We varied $c_{1}$ and $\mu_{B}=1-s$ in our simulations. For the simulations to estimate fixation probability, we ran 10^5^ independent simulations of the dynamics for each parameter set, where we ran the simulations until one of the genotypes fixed. We considered the major genotype as fixed when the minor genotype dropped below 0.1 in the population. We computed the fixation probability as simply the proportion of simulations where genotype A fixed in the population. Similarly for the simulations to estimate the site frequency spectra, we ran 2 · 10^4^ independent simulations of the dynamics for each parameter set. We recorded the genotype frequency at each timestep until one of the genotypes fixed, and the appropriately normalized the data to obtain the expected density of A at a given frequency bin.

## Supplementary Material

Supplement 1
