# Supplementary material for: Asynchronous abundance fluctuations can drive giant genotype frequency fluctuations
Source: bioRxiv. 2024 Aug 4:2024.02.23.581776. Preprint. [Version 3] doi: 10.1101/2024.02.23.581776 (PMC10983864; doi:10.1101/2024.02.23.581776)
Supplement: 1 [file NIHPP2024.02.23.581776V3-supplement-1.pdf]

bioRxiv preprint doi: https://doi.org/10.1101/2024.02.23.581776; this version posted August 4, 2024. The copyright holder for this preprint (which was not certified by peer review) is the author/funder, who has granted bioRxiv a license to display the preprint in perpetuity. It is made available under aCC-BY 4.0 International license.

List of Figures

S1 Fluctuations within the barcoded L library 15
S2 Coculture of REL606 and $\Delta pykF$ mutant 15
S3 Inferred power-law exponents 16
S4 Ratio of abundance fluctuation strengths between S and L 16
S5 Frequency-dependent fitness effects of S and L 17
S6 Abundance correlation 18
S7 Comparison of curve fits to overnight trajectory variance 19
S8 Cross-validation to compute Lyapunov exponent 19
S9 Rank correlation of replicates 20
S10 Frequency-dependent fitness effects of S and L 20
S11 Frequency variance scaling in batch 4 21
S12 MSD computed with different frequency range 21
S13 Effective frequency-dependent fitness effect 22
S14 The dependence of p_{fix} on s_e 23
S15 Site frequency spectrum with constant selection 24

bioRxiv preprint doi: https://doi.org/10.1101/2024.02.23.581776; this version posted August 4, 2024. The copyright holder for this preprint (which was not certified by peer review) is the author/funder, who has granted bioRxiv a license to display the preprint in perpetuity. It is made available under aCC-BY 4.0 International license.

S1 Table of strains

Strain Name	Internal Name	Ancestor	Fluorescent protein
REL606	eJA334		attTn7::sYFP2
REL606 $\Delta pykF$	eJA330	REL606	attTn7::mScarlet-I
6.5k $S1$	eJA246	REL11555	attTn7::eBFP2
6.5k $L1$	eJA245	REL11556	attTn7::sYFP2

S2 Effective model of population fluctuations

S2.1 Two-genotype case

To model the process of population fluctuations, we first consider a simple population consisting of one genotype, where all individuals are identical. The change in population size of a genotype μ from size N_μ in the current time point to size N'_μ in the next time point can be decomposed as

$$\Delta N_\mu \equiv N'_\mu - N_\mu = \sum_{i=1}^{N_\mu} n'_{\mu,i} - n_{\mu,i} \equiv \sum_{i=1}^{N_\mu} \Delta n_{\mu,i} \ , \tag{S1}$$

where $\Delta n_{\mu,i} \in [-1, 0, 1, \dots]$, $i \in [1, N_\mu]$ is -1 plus the offspring number of the ith cell of the N_μ cells of the μ genotype that exist at the current time step. We know that if all cells behave the same way, we have to demand

$$\langle \Delta n_{\mu,i} \Delta n_{\mu,j} \rangle = \begin{cases} c_{\mu,0} & \text{for } i = j \\ c_{\mu,1} & \text{for } i \neq j \end{cases} \tag{S2}$$

Here, the brackets $\langle\cdot\rangle$ represent the mean of a random variable. This is the only mathematical form of the covariance parameters that won't change if we relabel the cells–a symmetry argument that can easily be extended to multiple genotypes. This assumes that the offspring number distributions have finite covariances, i.e. are not heavy-tailed. Note that $c_1 < c_0$ follows from $0 < \langle(\Delta n_i - \Delta n_j)^2\rangle = 2c_0 - 2c_1$.

We can express the variance in total population size in terms of c_0 and c_1,

$$\text{var}(N'_\mu) = \text{var}(\Delta N_\mu) = \langle(\Delta N_\mu)^2\rangle \tag{S3}$$

$$= \left\langle \left(\sum_{i=1}^{N_\mu} \Delta n_{\mu,i} \right)^2 \right\rangle = \left\langle \sum_{i=1}^{N_\mu} \Delta n_{\mu,i}^2 + \sum_{i=1}^{N_\mu} \sum_{j\neq i}^{N_\mu} \Delta n_{\mu,i} \Delta n_{\mu,j} \right\rangle \tag{S4}$$

$$= (c_{\mu,0} - c_{\mu,1}) N_\mu + c_{\mu,1} N_\mu^2 \ . \tag{S5}$$

bioRxiv preprint doi: https://doi.org/10.1101/2024.02.23.581776; this version posted August 4, 2024. The copyright holder for this preprint (which was not certified by peer review) is the author/funder, who has granted bioRxiv a license to display the preprint in perpetuity. It is made available under aCC-BY 4.0 International license.

The form of this variance scaling has been previously noted [1, 2]. Power law mean-variance scaling of population abundance has been widely observed in ecology, where it is known as Taylor's power law [3, 4, 5, 6, 7]. For large enough values of N_μ, the distribution of N'_μ will converge in distribution to a gaussian random variable, because of the central limit theorem. So the fluctuations of N'_μ will solely depend on the variance, $\mathrm{var}(N'_\mu)$.

We can now extend the analysis to a population consisting of two genotypes labeled A and B, possibly with non-identical properties (Figure 2A). We can generalize the below analysis to multiple genotypes (SI section S2.3). Extending the symmetry argument from above, we now require five covariance parameters to fully describe the population,

$$\langle \Delta n_{\mu,i} \Delta n_{\nu,j} \rangle = \begin{cases} c_{0A} & \text{for } i = j \text{ and } \mu = \nu = A \\ c_{0B} & \text{for } i = j \text{ and } \mu = \nu = B \\ c_{1A} & \text{for } i \neq j \text{ and } \mu = \nu = A \\ c_{1B} & \text{for } i \neq j \text{ and } \mu = \nu = B \\ c_{AB} & \text{for } i \neq j \text{ and } \mu \neq \nu \end{cases} \tag{S6}$$

The expression for the variance of the total population size for each genotype is the same as in equation S5. The covariance of the total population sizes between genotypes is,

$$\mathrm{cov}(N'_A, N'_B) = c_{AB} N_A N_B = c_{AB} N_{tot}^2 f_A (1 - f_A) \; . \tag{S7}$$

Where $N_{tot} = N_A + N_B$ is the total population size, and $f_A = N_A/(N_A + N_B)$ is the frequency of genotype A in the population. The frequency of a genotype will also change from one time point to the next, induced by the fluctuations of individuals. The expected frequency variance at the next time point can be written as,

$$\mathrm{var}(f'_A) = \left\langle \left(\frac{\sum_{i=1}^{N_A} 1 + \Delta n_{A,i}}{\sum_{i=1}^{N_A} 1 + \Delta n_{A,i} + \sum_{j=1}^{N_B} 1 + \Delta n_{B,j}} - \frac{N_A}{N_{tot}} \right)^2 \right\rangle \tag{S8}$$

If we assume that the frequency deviations are small, we can expand in the deviations to obtain,

$$\mathrm{var}(f'_A) \approx f_A^2 \left\langle \left(\frac{\sum_{i=1}^{N_A} \Delta n_{A,i}}{N_A} - \frac{\sum_{i=1}^{N_A} \Delta n_{A,i} + \sum_{j=1}^{N_B} \Delta n_{B,j}}{N_{tot}} \right)^2 \right\rangle \tag{S9}$$

$$= \frac{f_A(1 - f_A)}{N_{tot}} \left[(c_{0A} - c_{1A})(1 - f_A) + (c_{0B} - c_{1B}) f_A \right] + \underbrace{(c_{1A} + c_{1B} - 2c_{AB})}_{=\delta} f_A^2 (1 - f_A)^2 \; . \tag{S10}$$

We can approximate the expected frequency by expanding to second order,

bioRxiv preprint doi: https://doi.org/10.1101/2024.02.23.581776; this version posted August 4, 2024. The copyright holder for this preprint (which was not certified by peer review) is the author/funder, who has granted bioRxiv a license to display the preprint in perpetuity. It is made available under aCC-BY 4.0 International license.

$$\langle f'_A \rangle \approx \frac{\langle N'_A \rangle}{\langle N'_{tot} \rangle} - \frac{\text{cov}\,(N'_A, N'_{tot})}{\langle N'_{tot} \rangle^2} + \frac{\text{var}\,(N'_{tot}) \langle N'_A \rangle}{\langle N'_{tot} \rangle^3} \tag{S11}$$

$$= f_A - \frac{f_A(1-f_A)}{N_{tot}} [(c_{0B} - c_{1B}) - (c_{0A} - c_{1A})] + c_{1B} f_A (1-f_A)^2 - c_{1A} f_A^2 (1-f_A) - c_{AB} f_A (1-f_A)(1-2f_A) \tag{S12}$$

S2.2 Fluctuating environments

We can incorporate the effects of a fluctuating environment into our model, decomposing offspring number fluctuations into intrinsic and extrinsic fluctuations. We consider a fluctuating environment that causes genotype fitness to fluctuate with gaussian noise, $w(E) \sim \mathcal{N}(s_0, v_E)$. Conditioned on the environmental state, the covariance matrix $\langle \Delta n_{\mu,i} \Delta n_{\nu,j} | E \rangle$ corresponds to equation S6, representing the intrinsic fluctuations of the system. And so the total abundance variance conditioned on the environmental state, $\text{var}(N'_\mu | E)$, also corresponds to equation S5.

To consider the combined effect of the intrinsic and extrinsic fluctuations on the total population abundance, we must marginalize out the effect of environmental fluctuations,

$$p(N'_\mu) = \int dw(E) p(N'_\mu | w(E)) p(w(E)) \ . \tag{S13}$$

The conditional abundance density will be gaussian by the central limit theorem,

$$p(N'_\mu | w(E)) = \mathcal{N}(N'_\mu, w(E) N_\mu, (c_{\mu,0} - c_{\mu,1}) N_\mu + c_{\mu,1} N_\mu^2) \ . \tag{S14}$$

Here, $\mathcal{N}(x, \mu, v)$ is a standard gaussian density. We use a standard identity for the product of gaussian densities,

$$\mathcal{N}(ax, \mu_1, v_1) N(x, \mu_2, v_2) = N(\mu_1, a\mu_2, v_1 + a^2 v_2) \mathcal{N}(x, a\mu_{12}, a^2 v_{12})$$
$$\mu_{12}(\mu_1, \mu_2, v_1, v_2) = \frac{\mu_1 v_2 + \mu_2 v_1}{v_1 + v_2}$$
$$v_{12}(v_1, v_2) = \frac{1}{1/v_1 + 1/v_2}$$

By rearranging, applying the above identity, and integrating equation S13, we obtain a final marginal density of

$$\text{var}(N'_\mu) = (c_{\mu,0} - c_{\mu,1}) N_\mu + (c_{\mu,1} + v_E) N_\mu^2 \ . \tag{S15}$$

Thus, the total strength of correlated abundance fluctuations is simply the sum of the strength of fitness fluctuations (extrinsic source) and the strength of the intrinsic fluctuations. Applying the same logic, it immediately follows that the decoupling parameter δ can also be decomposed into intrinsic and extrinsic components, $\delta \to \delta_I + \delta_E$.

bioRxiv preprint doi: https://doi.org/10.1101/2024.02.23.581776; this version posted August 4, 2024. The copyright holder for this preprint (which was not certified by peer review) is the author/funder, who has granted bioRxiv a license to display the preprint in perpetuity. It is made available under aCC-BY 4.0 International license.

S2.3 Generalization to m genotypes

When there are m distinct genotypes/species present in a population, possibly with differing offspring number covariances, what is the expected frequency variance?

We first consider the possible covariance parameters between the change in offspring number for a single individual across a single generation, $\Delta n_{\mu,i}$, where μ labels the genotype and i labels the individual. Again, $\Delta n_{\mu,i} \in [-1, 0, 1, \dots]$, $i \in [1, N_\mu]$ is -1 plus the offspring number of the ith cell of the N_μ cells of the μ genotype that exist at the current time step. For m genotypes, we require $2m + m(m-1)/2$ covariance parameters:

$$\langle \Delta n_{\mu,i} \Delta n_{\nu,j} \rangle = \begin{cases} c_{0\mu} & \text{for } i = j \text{ and } \mu = \nu \\ c_{1\mu} & \text{for } i \neq j \text{ and } \mu = \nu \\ c_{\mu\nu} & \text{for } i \neq j \text{ and } \mu \neq \nu \ . \end{cases} \tag{S16}$$

We define the initial frequency of genotype A (arbitrarily labeled) as,

$$f_A = \frac{N_A}{N_{tot}} \ , \tag{S17}$$

where $N_{tot} = \sum_\mu^m N_\mu$. The frequency after one generation is similarly defined, and labeled as f'_A. If we assume that frequency deviations are small, we can expand in the deviations to obtain,

$$\text{var}(f'_A) \approx f_A^2 \left\langle \left(\frac{\sum_{i=1}^{N_A} \Delta n_{A,i}}{N_A} - \frac{\sum_\mu^m \sum_{i=1}^{N_\mu} \Delta n_{\mu,i}}{N_{tot}} \right)^2 \right\rangle \ . \tag{S18}$$

Following some algebra, and using equation S16, we arrive at the following expression,

$$\begin{aligned} \text{var}(f'_A) \approx\ & f_A(1-f_A)^2 \frac{c_{0A} - c_{1A}}{N_{tot}} + \frac{f_A^2}{N_{tot}} \sum_{\mu \neq A} (c_{0\mu} - c_{1\mu}) f_\mu \\ & + c_{1A} f_A^2 (1-f_A)^2 + f_A^2 \sum_{\mu \neq A} c_{1\mu} f_\mu^2 \\ & - 2 f_A^2 (1-f_A) \sum_{\mu \neq A} c_{A\mu} f_\mu + 2 f_A^2 \sum_{\mu \neq A} \sum_{\nu \neq A} c_{\mu\nu} f_\mu f_\nu \ . \end{aligned} \tag{S19}$$

We can see that the generalized equation has a similar form to the two-species case, where the first two terms arise from independent offspring number fluctuations, and the remaining terms arise from correlated offspring number fluctuations.

bioRxiv preprint doi: https://doi.org/10.1101/2024.02.23.581776; this version posted August 4, 2024. The copyright holder for this preprint (which was not certified by peer review) is the author/funder, who has granted bioRxiv a license to display the preprint in perpetuity. It is made available under aCC-BY 4.0 International license.

S3 Evolutionary implications

To study the implications of decoupling noise, we consider our two-genotype model in the continuous time diffusion limit under constant selection (assuming small deviations in total population size),

$$\begin{aligned}\partial_t f &= \left[s + \kappa_B - \kappa_A - c_{1A} f + c_{1B}(1-f) - c_{AB}(1-2f)\right] f(1-f) \\ &\quad + \sqrt{f(1-f)\left[\kappa_A(1-f) + \kappa_B f\right] + \delta f^2 (1-f)^2}\,\eta(t)\ .\end{aligned} \tag{S20}$$

Where $\eta(t)$ is standard gaussian white noise, $\langle \eta(t) \rangle = 0$ and $\langle \eta(t)\eta(t')\rangle = \delta(t-t')$. We use the approximate frequency mean and variance for the drift and diffusion terms from equations S12 and S10, respectively. We have combined several parameters for notational simplicity,

$$\kappa_A = (c_{0A} - c_{1A})/N_{tot} \tag{S21}$$

$$\kappa_B = (c_{0B} - c_{1B})/N_{tot}\ . \tag{S22}$$

This model is similar to those analyzed in previous studies. Gillespie (1974)[8] studied the case where $\delta = 0$ but $\kappa_A \neq \kappa_B$. Conversely, Melbinger and Vergassola (2015)[9] studied the case where $\delta \neq 0$ but $\kappa_A = \kappa_B$.

S3.1 Fixation probability

Given that the genotype/mutant gets introduced into the population at some initial frequency f_0, what is the probability that it will fix in the population? To find this, we'll use the Kolmogorov Backward Equation (KBE) for this system. In general, the KBE is,

$$\frac{d\Pi}{dt} = v(f)\frac{d\Pi}{df} + D(f)\frac{d^2\Pi}{df^2} \tag{S23}$$

In this case, the drift and diffusion terms are given by,

$$v(f) = \left[s - \kappa_A + \kappa_B - c_{1A} f + c_{1B}(1-f) - c_{AB}(1-2f)\right] f(1-f) \tag{S24}$$

$$D(f) = \frac{1}{2}\left[f(1-f)\left(\kappa_A(1-f) + \kappa_B f\right) + \delta f^2 (1-f)^2\right] \tag{S25}$$

The steady state solution of equation S23 gives us the fraction of trajectories that end up at each of the absorbing states (here: 0 or 1). After setting equation S23 to 0, and applying the boundary conditions $\Pi(0) = 0$ and $\Pi(1) = 1$, we can rearrange and integrate to obtain a general solution for the fixation probability,

bioRxiv preprint doi: https://doi.org/10.1101/2024.02.23.581776; this version posted August 4, 2024. The copyright holder for this preprint (which was not certified by peer review) is the author/funder, who has granted bioRxiv a license to display the preprint in perpetuity. It is made available under aCC-BY 4.0 International license.

$$\pi(f) = \int df e^{-\int df v(f)/D(f)} \tag{S26}$$

$$p_{fix}(f_0) = \frac{\pi(f_0) - \pi(0)}{\pi(1) - \pi(0)} . \tag{S27}$$

For our system, the fixation probability becomes,

$$p_{fix}(f_0) = \frac{A(f_0)^{2s_e/\beta} - A_0^{2s_e/\beta}}{A_1^{2s_e/\beta} - A_0^{2s_e/\beta}} \tag{S28}$$

$$A(f_0) = \left(\frac{\beta + \delta(1-2f_0) - \Delta\kappa}{\beta - \delta(1-2f_0) + \Delta\kappa}\right) \tag{S29}$$

$$A_0 = A(0) \tag{S30}$$

$$A_1 = A(1) \tag{S31}$$

Or expanded,

$$p_{fix}(f_0) = \frac{\left(\frac{\beta+\delta(1-2f_0)-\Delta\kappa}{\beta-\delta(1-2f_0)+\Delta\kappa}\right)^{2s_e/\beta} - \left(\frac{\beta+\delta-\Delta\kappa}{\beta-\delta+\Delta\kappa}\right)^{2s_e/\beta}}{\left(\frac{\beta-\delta-\Delta\kappa}{\beta+\delta+\Delta\kappa}\right)^{2s_e/\beta} - \left(\frac{\beta+\delta-\Delta\kappa}{\beta-\delta+\Delta\kappa}\right)^{2s_e/\beta}} \tag{S32}$$

Which are in terms of new compound parameters,

$$\beta = \sqrt{\Delta\kappa^2 + \delta(\delta + 2(\kappa_A + \kappa_B))} \tag{S33}$$

$$s_e = s + \frac{1}{2}(c_{1B} - c_{1A} - \Delta\kappa) \tag{S34}$$

$$\Delta\kappa = \kappa_A - \kappa_B \tag{S35}$$

We are typically interested in the case where the initial frequency of the minor genotype is small, perhaps because it arose via spontaneous mutation in the population, or an individual with that genotype migrated to the population. Thus, we would like to focus on the limit of small initial frequency, $f_0 \ll 1$. We start by Taylor expanding equation S29 around $f_0 = 0$,

$$A(f_0) \approx A_0^{2s_e/\beta} - \frac{8f_0\delta s_e}{(\beta-\delta+\Delta\kappa)^2}A_0^{2s_e/\beta-1} + \frac{16f_0^2\delta^2 s_e(\delta - \Delta\kappa + 2s_e)}{(\beta-\delta+\Delta\kappa)^2(\beta+\delta-\Delta\kappa)^2}A_0^{2s_e/\beta} + O(f_0^3) . \tag{S36}$$

bioRxiv preprint doi: https://doi.org/10.1101/2024.02.23.581776; this version posted August 4, 2024. The copyright holder for this preprint (which was not certified by peer review) is the author/funder, who has granted bioRxiv a license to display the preprint in perpetuity. It is made available under aCC-BY 4.0 International license.

Following some algebra, we see that the second-order term in the expansion is negligible compared to the first-order term when,

$$f_0 \ll 2\kappa_A \left[\delta + 2s_e - \Delta\kappa\right]^{-1} . \tag{S37}$$

In this limit, we can truncate equation S36 to first order in f_0; the fixation probability becomes,

$$p_{fix}(f_0) \approx \frac{2 f_0 s_e A_0^{2s_e/\beta}}{\kappa_A \left(A_0^{2s_e/\beta} - A_1^{2s_e/\beta}\right)} . \tag{S38}$$

With this expression, we now wish to analyze the behavior of p_{fix} in the limit of weak selection, $s_e \to 0$. We thus expand in s_e,

$$p_{fix}(f_0) \approx \frac{f_0}{\kappa_A}\left(\frac{\beta}{\log A_0 - \log A_1} + \frac{s_e}{2}\right) + O(s_e^2) . \tag{S39}$$

By comparing the first two terms, we see that p_{fix} is approximately constant at low s_e,

$$p_{fix}(f_0) \approx \frac{f_0 \beta}{\kappa_A \left(\log A_0 - \log A_1\right)} . \tag{S40}$$

This approximation is valid when $s_e \ll s_e^*$, where,

$$s_e^* = \frac{\beta}{\log A_0 - \log A_1} \tag{S41}$$

We recover the classical expression for the critical fitness effect when decoupling noise is weak, $\delta \ll \kappa$, and when $\Delta\kappa = 0$,

$$s_e^* \approx 1/N_e , \tag{S42}$$

and thus we also see that the fixation probability when $s_e \ll s_e^*$ is simply f_0 in this case.

Now we turn to analyzing the opposite limit, where selection is strong. We will go back to the unapproximated expression for p_{fix} in equation S32. Rearranging, we obtain,

bioRxiv preprint doi: https://doi.org/10.1101/2024.02.23.581776; this version posted August 4, 2024. The copyright holder for this preprint (which was not certified by peer review) is the author/funder, who has granted bioRxiv a license to display the preprint in perpetuity. It is made available under aCC-BY 4.0 International license.

$$p_{fix}(f_0) = \frac{1 - \left(\frac{A(f_0)}{A_0}\right)^{2s_e/\beta}}{1 - \left(\frac{A_1}{A_0}\right)^{2s_e/\beta}} \tag{S43}$$

$$= \frac{1 - e^{-\frac{2s_e}{\beta}[\log A_0 - \log A(f_0)]}}{1 - e^{-\frac{2s_e}{\beta}[\log A_0 - \log A_1]}} \,. \tag{S44}$$

When $s_e \gg s_e^*$ and $\Delta\kappa = 0$, we can expand in either small δ or small N_e to recover Kimura's classical fixation probability [10],

$$p_{fix}(f_0) \approx \frac{1 - e^{-2s_e N_e f_0}}{1 - e^{-2s_e N_e}} \tag{S45}$$

This approximation is also valid in the limit of weak decoupling noise, $\delta \ll \frac{6\kappa}{f_0(2f_0-3)}$.

S3.2 Site frequency spectrum

The site frequency spectrum (SFS) describes the expected density of derived alleles at a given frequency; specifically $p_{SFS}(f)df$ is the number of derived alleles in the frequency range $[f - df/2, f + df/2]$ [11]. We calculate the SFS for alleles affected by decoupling noise by leveraging previously described approaches [12, 13] along with our Langevin equation (equation S20). We find a general closed form solution for the SFS in terms of the frequency of alleles with parameters from A,

$$p_{SFS}(f_A) = \theta \frac{\phi(f_A) - \phi(1)}{(1 - \phi(1))(1 - f_A) f_A} \tag{S46}$$

$$\phi(f_A) = \left(\frac{(\beta + \delta - \Delta\kappa)(\beta - \delta(1 - 2f_A) + \Delta\kappa)}{(\beta - \delta + \Delta\kappa)(\beta + \delta(1 - 2f_A) - \Delta\kappa)}\right)^{2s_e/\beta} \,. \tag{S47}$$

When $f = 0.5$ and $\Delta\kappa = 0$, the SFS reduces to,

$$p_{SFS}(0.5) = 4\theta \frac{\left(\frac{\beta-\delta}{\beta+\delta}\right)^{2s_e/\beta} - 1}{\left(\frac{\beta-\delta}{\beta+\delta}\right)^{4s_e/\beta} - 1} \,. \tag{S48}$$

In the limit that $\delta \gg 1/N_e$,

$$\frac{\beta + \delta}{\beta - \delta} \approx \delta N_e \,. \tag{S49}$$

bioRxiv preprint doi: https://doi.org/10.1101/2024.02.23.581776; this version posted August 4, 2024. The copyright holder for this preprint (which was not certified by peer review) is the author/funder, who has granted bioRxiv a license to display the preprint in perpetuity. It is made available under aCC-BY 4.0 International license.

and,

$$p_{SFS}(0.5) \approx \frac{4\theta}{1+(\delta N_e)^{-2s_e/\delta}} \ . \tag{S50}$$

Similarly, the limit of the SFS as $f \to 1$ is,

$$p_{SFS}(1) = \frac{8\delta s_e}{(\beta-\delta)(\beta+\delta)\left(1-\left(\frac{\beta-\delta}{\beta+\delta}\right)^{4s_e/\beta}\right)} \ . \tag{S51}$$

Applying the limit that $\delta \gg 1/N_e$,

$$p_{SFS}(1) \approx \frac{2s_e N_e \theta}{1-(\delta N_e)^{-4s_e/\delta}} \ . \tag{S52}$$

If we are interested in the weak selection limit, where $s \to 0$, and again $\Delta\kappa = 0$ then equation S46 becomes,

$$p_{SFS} = \frac{\theta}{2f_A(1-f_A)} \frac{\log\left[\frac{(1-f_A)(\delta-\beta)+2/N_e}{(1-f_A)(\delta+\beta)+2/N_e}\right]}{\log\left[\frac{2/N_e}{\delta+\beta+2/N_e}\right]} \ . \tag{S53}$$

In the limit of strong decoupling noise, $\delta \gg N_e$, equation S53 becomes,

$$p_{SFS} \approx \frac{\theta}{2f_A(1-f_A)\log(\delta N_e)} \log\left[\frac{(1-f_A)\delta+1/N_e}{f_A/N_e}\right] \ . \tag{S54}$$

In the high frequency limit, $(1-f) \ll 1$,

$$p_{SFS} \approx \theta \frac{\log\left[(1-f_A)\delta N_e + 1\right]}{2(1-f_A)\log(\delta N_e)} \ . \tag{S55}$$

We see that when we additionally apply the limit that $(1-f_A) \gg (\delta N_e)^{-1}$, we see that the SFS shows an uptick in the intermediate-high frequency regime,

$$p_{SFS} \approx \theta \frac{1}{2(1-f_A)} \left(\frac{\log(1-f_A)}{\log(\delta N_e)} + 1\right) \ . \tag{S56}$$

We find that in the limit of weak correlated fluctuations, $\delta \ll 1/N_e$, and when $\kappa_A = \kappa_B$, equation S46 reduces to the classical expression [14] for the site frequency spectrum for mutations under constant selection, i.e.

bioRxiv preprint doi: https://doi.org/10.1101/2024.02.23.581776; this version posted August 4, 2024. The copyright holder for this preprint (which was not certified by peer review) is the author/funder, who has granted bioRxiv a license to display the preprint in perpetuity. It is made available under aCC-BY 4.0 International license.

$$p_{SFS} = \theta \frac{e^{2sN_e}(1 - e^{-2s(1-f_A)N_e})}{(e^{2sN_e} - 1) f_A (1 - f_A)} \,. \tag{S57}$$

S4 Chaotic dynamics

S4.1 Variance between biological replicates

Here we show how exponentially increasing variance between biological replicates is equivalent to exponentially diverging trajectories, which is characteristic of chaotic dynamics. Specifically, in a one-dimensional system where we observe two trajectories, f_i and f_j, initially separated by ϵ, we expect that at small times, their distance from each other will grow exponentially,

$$|f_i(t) - f_j(t)| = \epsilon_{i,j} e^{\lambda t} \,. \tag{S58}$$

In chaotic systems, $\lambda > 0$. If we observe N initially nearby trajectories, labeled f_i (dropping the time index for simplicity), then the variance between trajectories is,

$$\begin{aligned}
\operatorname{var} f(t) &= N^{-1} \sum_i \left(f_i - \frac{1}{N} \sum_j f_j \right)^2 && \text{(S59)} \\
&= N^{-3} \sum_i \left(N f_i - \sum_j f_j \right)^2 && \text{(S60)} \\
&= N^{-3} \sum_i \left[N^2 f_i^2 - 2N f_i \sum_j f_j + \left(\sum_j f_j \right)^2 \right] && \text{(S61)} \\
&= N^{-3} \left[N^2 \sum_i f_i^2 - N \left(\sum_i f_i^2 + 2 \sum_i \sum_{j>i} f_i f_j \right) \right] && \text{(S62)} \\
&= N^{-2} \left[(N-1) \sum_i f_i^2 - 2 \sum_i \sum_{j>i} f_i f_j \right] && \text{(S63)} \\
&= N^{-2} \left[\sum_i \sum_{j>i} (f_i - f_j)^2 \right] \,. && \text{(S64)}
\end{aligned}$$

From equation S58, we can then replace $(f_i - f_j)^2$ with $\epsilon_{i,j} e^{2\lambda t}$, obtaining,

bioRxiv preprint doi: https://doi.org/10.1101/2024.02.23.581776; this version posted August 4, 2024. The copyright holder for this preprint (which was not certified by peer review) is the author/funder, who has granted bioRxiv a license to display the preprint in perpetuity. It is made available under aCC-BY 4.0 International license.

$$\operatorname{var} f(t) = \operatorname{var} f(0)\, e^{2\lambda t} \tag{S65}$$

References

[1] M. S. Bartlett. Some Notes on Insecticide Tests in the Laboratory and in the Field. *Supplement to the Journal of the Royal Statistical Society*, 3(2):185, 1936.

[2] Ford Ballantyne IV and Andrew J. Kerkhoff. The observed range for temporal mean-variance scaling exponents can be explained by reproductive correlation. *Oikos*, 116(1):174–180, 2007. _eprint: https://onlinelibrary.wiley.com/doi/pdf/10.1111/j.2006.0030-1299.15383.x.

[3] L. R. Taylor. Aggregation, Variance and the Mean. *Nature*, 189(4766):732–735, March 1961. Number: 4766 Publisher: Nature Publishing Group.

[4] Matthew R. D. Cobain, Markus Brede, and Clive N. Trueman. Taylor's power law captures the effects of environmental variability on community structure: An example from fishes in the North Sea. *Journal of Animal Ecology*, 88(2):290–301, 2019. _eprint: https://onlinelibrary.wiley.com/doi/pdf/10.1111/1365-2656.12923.

[5] Joel E. Cohen and Meng Xu. Random sampling of skewed distributions implies Taylor's power law of fluctuation scaling. *Proceedings of the National Academy of Sciences*, 112(25):7749–7754, June 2015. Publisher: Proceedings of the National Academy of Sciences.

[6] Meng Xu. Taylor's power law: before and after 50 years of scientific scrutiny, April 2016. arXiv:1505.02033 [q-bio].

[7] Zoltán Eisler, Imre Bartos, and János Kertész. Fluctuation scaling in complex systems: Taylor's law and beyond1. *Advances in Physics*, 57(1):89–142, January 2008.

[8] John H. Gillespie. Natural Selection for within-Generation Variance in Offspring Number. *Genetics*, 76(3):601–606, March 1974.

[9] Anna Melbinger and Massimo Vergassola. The Impact of Environmental Fluctuations on Evolutionary Fitness Functions. *Scientific Reports*, 5(1):15211, October 2015. Number: 1 Publisher: Nature Publishing Group.

[10] M. Kimura. On the probability of fixation of mutant genes in a population. *Genetics*, 47(6):713–719, June 1962.

[11] Motoo Kimura. The Number of Heterozygous Nucleotide Sites Maintained in a Finite Population Due to Steady Flux of Mutations. *Genetics*, 61(4):893–903, April 1969.

[12] R. C. Griffiths. The frequency spectrum of a mutation, and its age, in a general diffusion model. *Theoretical Population Biology*, 64(2):241–251, September 2003.

[13] Steven N. Evans, Yelena Shvets, and Montgomery Slatkin. Non-equilibrium theory of the allele frequency spectrum. *Theoretical Population Biology*, 71(1):109–119, February 2007.

bioRxiv preprint doi: https://doi.org/10.1101/2024.02.23.581776; this version posted August 4, 2024. The copyright holder for this preprint (which was not certified by peer review) is the author/funder, who has granted bioRxiv a license to display the preprint in perpetuity. It is made available under aCC-BY 4.0 International license.

[14] Warren J. Ewens. *Mathematical Population Genetics 1: Theoretical Introduction*. Springer Science & Business Media, January 2004.

[15] Joao A. Ascensao, Kelly M. Wetmore, Benjamin H. Good, Adam P. Arkin, and Oskar Hallatschek. Quantifying the local adaptive landscape of a nascent bacterial community. *Nature Communications*, 14(1):248, January 2023. Number: 1 Publisher: Nature Publishing Group.

[16] Daniel E Rozen and Richard E Lenski. Long-term experimental evolution in escherichia coli. viii. dynamics of a balanced polymorphism. *The American Naturalist*, 155(1):24–35, 2000.

[17] Joao A. Ascensao, Jonas Denk, Kristen Lok, QinQin Yu, Kelly M. Wetmore, and Oskar Hallatschek. Rediversification following ecotype isolation reveals hidden adaptive potential. *Current Biology*, 34(4):855–867.e6, February 2024.

[18] Michael T. Rosenstein, James J. Collins, and Carlo J. De Luca. A practical method for calculating largest Lyapunov exponents from small data sets. *Physica D: Nonlinear Phenomena*, 65(1-2):117–134, May 1993.

[19] Sandeep Venkataram, Barbara Dunn, Yuping Li, Atish Agarwala, Jessica Chang, Emily R. Ebel, Kerry Geiler-Samerotte, Lucas Hérissant, Jamie R. Blundell, Sasha F. Levy, Daniel S. Fisher, Gavin Sherlock, and Dmitri A. Petrov. Development of a Comprehensive Genotype-to-Fitness Map of Adaptation-Driving Mutations in Yeast. *Cell*, 166(6):1585–1596.e22, September 2016.

bioRxiv preprint doi: https://doi.org/10.1101/2024.02.23.581776; this version posted August 4, 2024. The copyright holder for this preprint (which was not certified by peer review) is the author/funder, who has granted bioRxiv a license to display the preprint in perpetuity. It is made available under aCC-BY 4.0 International license.

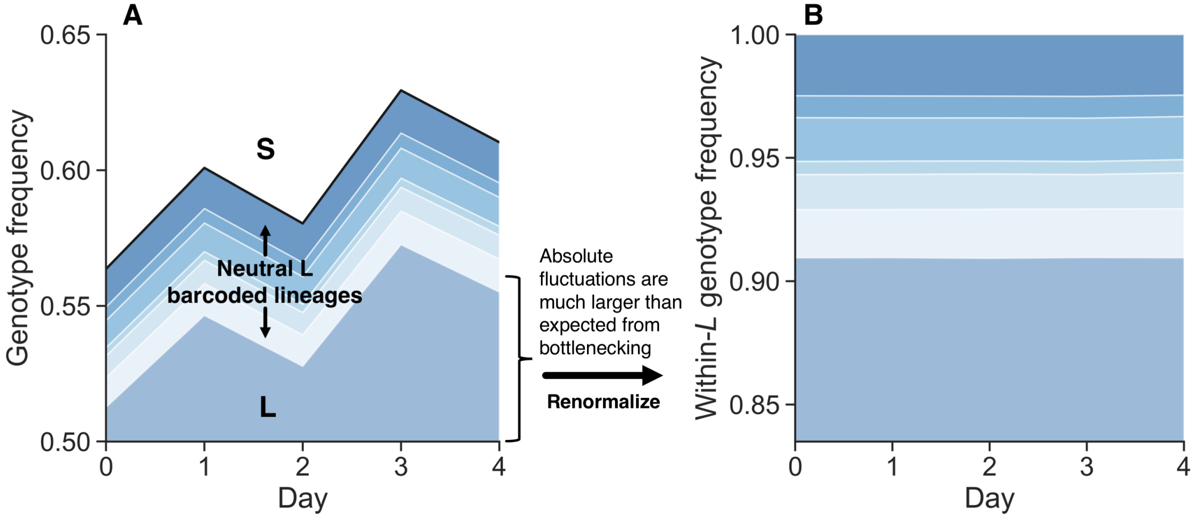

Figure S1: Fluctuations within the barcoded L library and relative to S (related to Figure 1A-B). (**A, B**) Randomly barcoded libraries of *E. coli* strains S and L were propagated together in their native serial dilution environment (previously reported data [15]). In the muller plot representation of lineage sizes, we see that the total frequency of L relative to S shows large fluctuations. However, neutral barcoded lineages within L show substantially smaller fluctuations relative to each other.

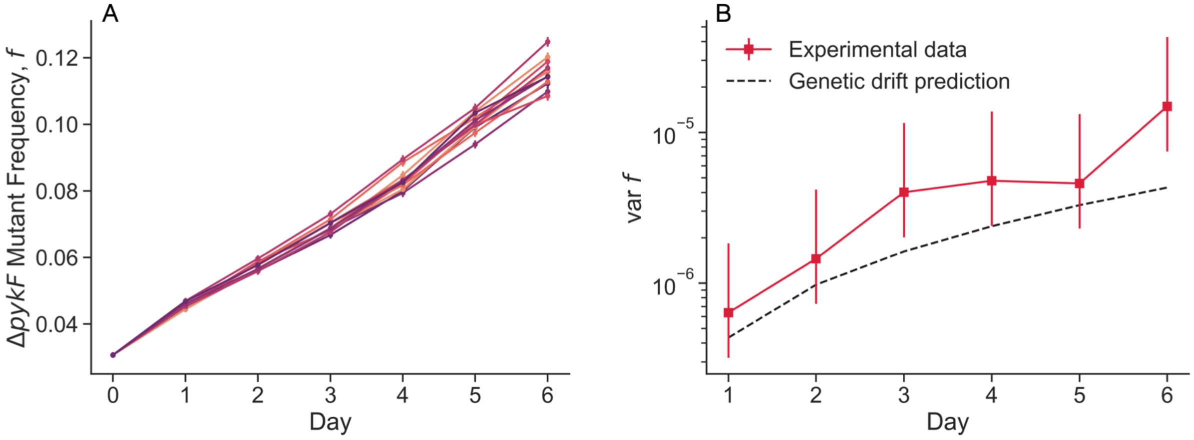

Figure S2: Coculture of REL606 and $\Delta pykF$ mutant (related to Figure 1D-E). (**A**) Cocultures were split at day 0 into 8 biological replicates, then propagated separately in the same environment. Flow cytometry measurements were taken every day. (**B**) We computed the variance between biological replicates, and compared it to the expectation that classical genetic drift is the sole source of population stochasticity, assuming $N_e = 10^5$. Error bars represent 95% CIs.

bioRxiv preprint doi: https://doi.org/10.1101/2024.02.23.581776; this version posted August 4, 2024. The copyright holder for this preprint (which was not certified by peer review) is the author/funder, who has granted bioRxiv a license to display the preprint in perpetuity. It is made available under aCC-BY 4.0 International license.

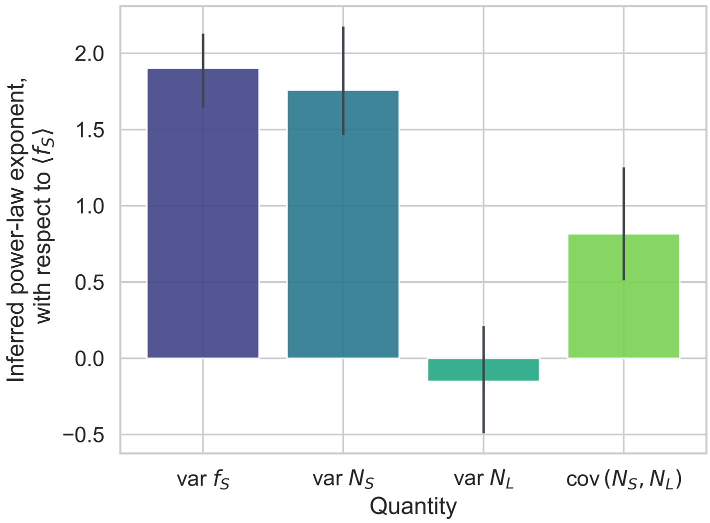

Figure S3: Inferred power-law exponents for variance and covariance quantities, as a function of mean S frequency (related to Figure 2). Error bars represent 95% confidence intervals, computed via bootstrapping.

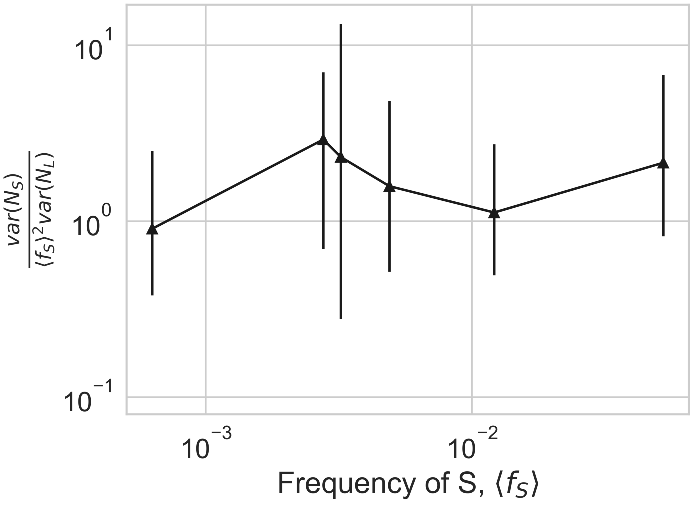

Figure S4: Ratio of abundance fluctuation strengths between S and L, including the f^2 factor for S (related to Figure 2). The ratio is equal to c_{1S}/c_{1L} under our model. We see that the ratio is a small factor of order 1, indicating that S and L fluctuate by approximately the same amount. Error bars represent 95% confidence intervals.

bioRxiv preprint doi: https://doi.org/10.1101/2024.02.23.581776; this version posted August 4, 2024. The copyright holder for this preprint (which was not certified by peer review) is the author/funder, who has granted bioRxiv a license to display the preprint in perpetuity. It is made available under aCC-BY 4.0 International license.

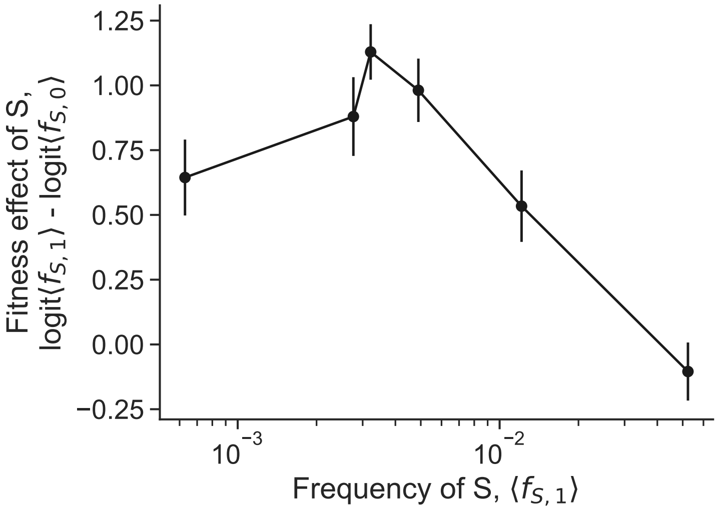

Figure S5: Frequency-dependent fitness effects of S and L (related to Figure 2). We computed the fitness effect of S relative to L across frequency. Consistent with prior measurements [16, 17], we find negative frequency-dependent fitness effects. Error bars represent 95% confidence intervals.

bioRxiv preprint doi: https://doi.org/10.1101/2024.02.23.581776; this version posted August 4, 2024. The copyright holder for this preprint (which was not certified by peer review) is the author/funder, who has granted bioRxiv a license to display the preprint in perpetuity. It is made available under aCC-BY 4.0 International license.

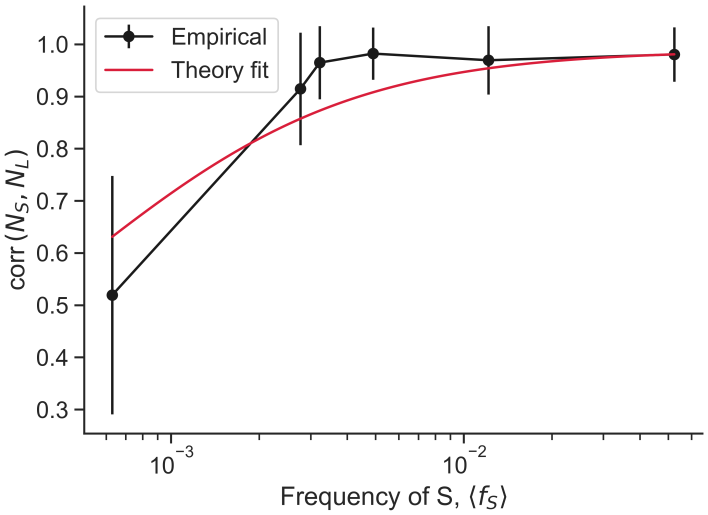

Figure S6: Correlation between the abundance of S and L (related to Figure 2). Our model predicts that the correlation between abundance should be constant when decoupling noise is dominant ($\rho_{AB} = c_{AB}/\sqrt{c_{1A}c_{1B}}$). But once classical genetic drift becomes non-negligible, the correlation should decline; this perhaps indicates that the lowest data point is around the cross-over between the drift- and decoupling-dominated regimes. We fit the theoretical curve to our experimental data via ordinary least squares regression. Error bars are standard errors.

bioRxiv preprint doi: https://doi.org/10.1101/2024.02.23.581776; this version posted August 4, 2024. The copyright holder for this preprint (which was not certified by peer review) is the author/funder, who has granted bioRxiv a license to display the preprint in perpetuity. It is made available under aCC-BY 4.0 International license.

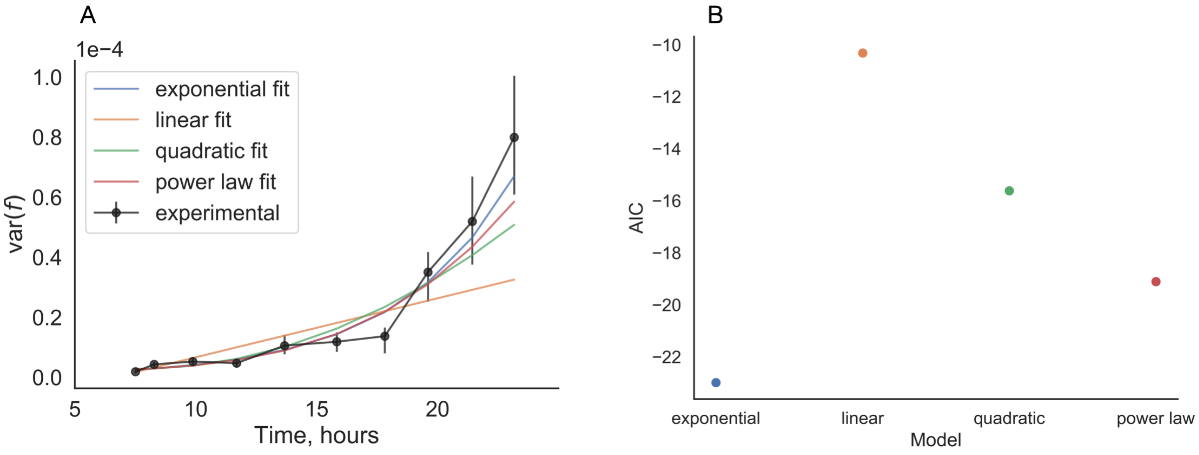

Figure S7: Comparison of curve fits to the variance of overnight frequency trajectories (related to Figure 4). (**A**) Best fits of various curves to the experimental variance measurements. (**B**) Akaike Information Criterion (AIC) of fitted models to experimental data, to evaluate goodness-of-fit. We computed FDR-corrected p-values to evaluate if the difference between the AIC of the exponential model compared to the other fits was signficant. The p-values were p =0.018, 0.058, and 0.022 for the comparison to the linear, quadratic, power law models respectively.

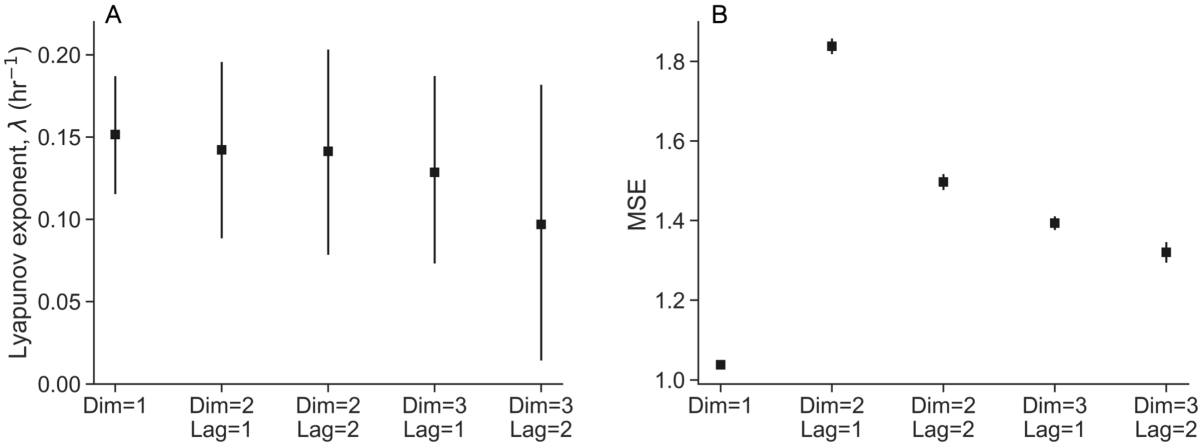

Figure S8: Cross-validation to compute Lyapunov exponent (related to Figure 4). We embedded the overnight trajectories in dimensions 1-3 by using a lag of 1-2 time points. (**A**) We computed the Lyapunov exponents using the method of Rosenstein et al. [18] for all conditions. (**B**) In order to choose which set of dimension and lag hyperparameters were most suitable for our data, we performed shuffle splitting cross-validation, and computed an out-of-sample mean squared error (MSE) for all conditions. All error bars represent 95% confidence intervals.

bioRxiv preprint doi: https://doi.org/10.1101/2024.02.23.581776; this version posted August 4, 2024. The copyright holder for this preprint (which was not certified by peer review) is the author/funder, who has granted bioRxiv a license to display the preprint in perpetuity. It is made available under aCC-BY 4.0 International license.

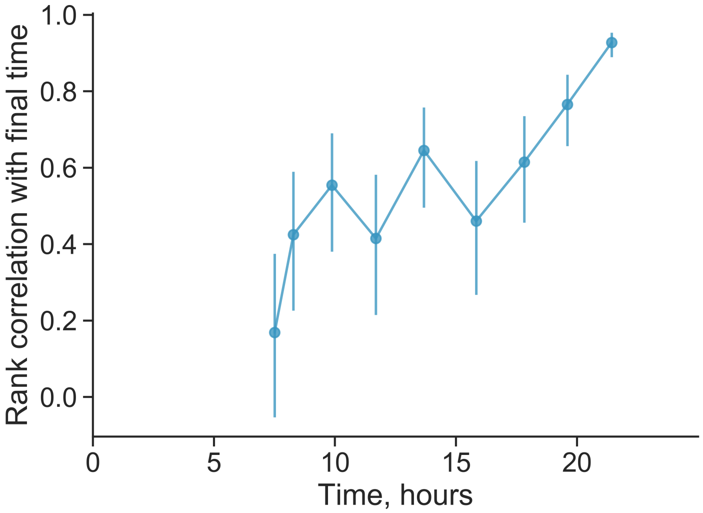

Figure S9: We quantified the rank correlation of replicates with its rank at the final time (related to Figure 4). The increase in correlation in the last 7 hours further shows how the decoupling noise accumulates over time, and that the rank position of each replicate is not "frozen in" earlier in the time course.

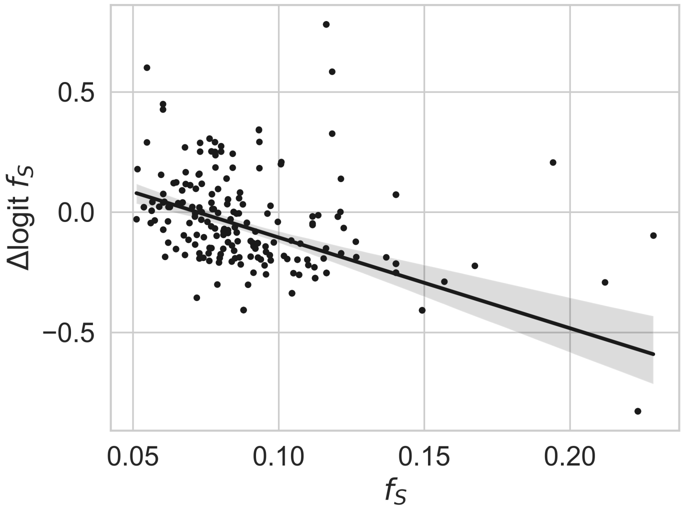

Figure S10: Frequency-dependent fitness effects of S and L (related to Figure 5). Scatter points represent change in logit frequency across a single day. Fitted line represents marginal frequency-dependent effect from the hierarchical model, described in the main text.

bioRxiv preprint doi: https://doi.org/10.1101/2024.02.23.581776; this version posted August 4, 2024. The copyright holder for this preprint (which was not certified by peer review) is the author/funder, who has granted bioRxiv a license to display the preprint in perpetuity. It is made available under aCC-BY 4.0 International license.

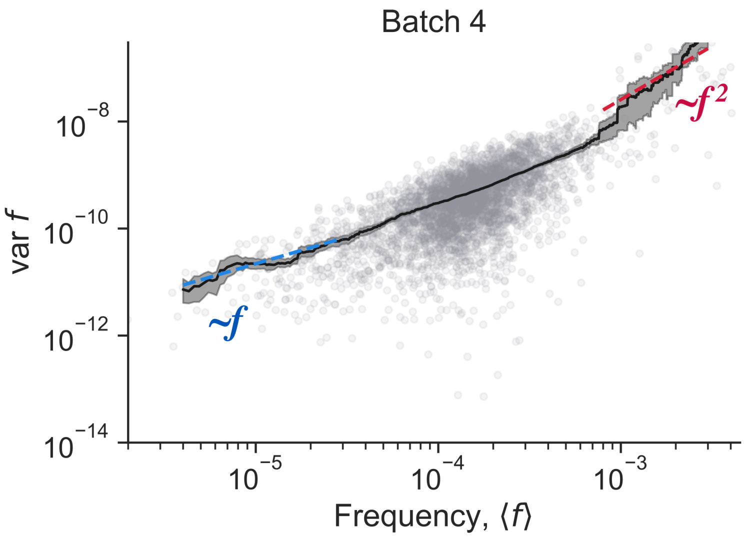

Figure S11: Frequency variance scaling in batch 4 in Venkataram et al.[19] data (related to Figure 6). Colored dashed lines represent the indicated scaling. Unconstrained power-law fits yields power law exponents of 2.15 ± 0.13 at high frequencies and 1.03 ± 0.08 at low frequencies.

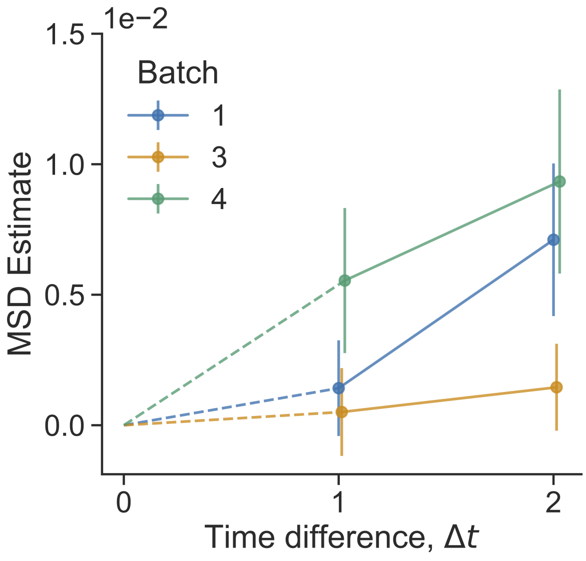

Figure S12: Mean squared displacement estimate computed with different frequency range, from Venkataram et al.[19] data (related to Figure 6B). Here, we focused on barcodes from a thinner mean frequency range (compared to the main text), $7 \cdot 10^{-4}$ to $2 \cdot 10^{-3}$. As there were fewer barcodes in this range, the estimates are noisier. Error bars represent 95% CIs.

bioRxiv preprint doi: https://doi.org/10.1101/2024.02.23.581776; this version posted August 4, 2024. The copyright holder for this preprint (which was not certified by peer review) is the author/funder, who has granted bioRxiv a license to display the preprint in perpetuity. It is made available under aCC-BY 4.0 International license.

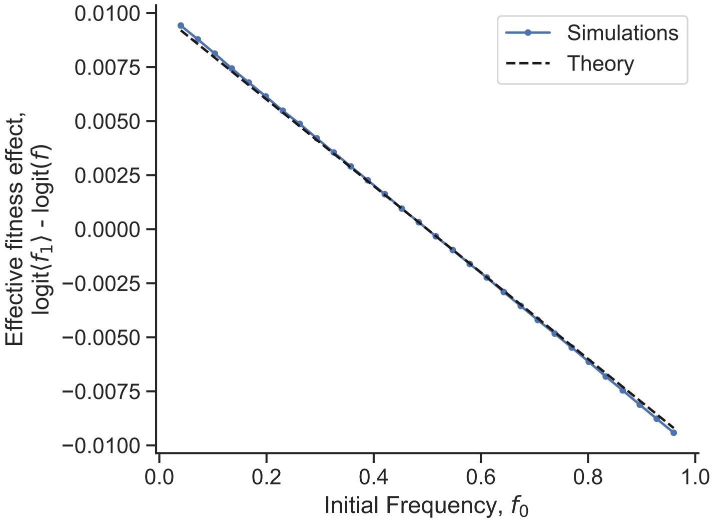

Figure S13: Effective frequency-dependent fitness effect induced by decoupling fluctuations (related to Figure 7). We simulated two uncorrelated population sizes with no selective difference, but that experience both genetic drift and decoupling noise, $N_{A,1} \sim \mathcal{N}\left(N_{A,0}, (c_{0A} - c_{1A})N_{A,0} + c_{1A}N_{A,0}^2\right)$ and $N_{B,1} \sim \mathcal{N}\left(N_{B,0}, (c_{0B} - c_{1B})N_{B,0} + c_{1B}N_{B,0}^2\right)$. In the simulations, we used $c_{0A} = c_{0B} = 1$ and $c_{1A} = c_{1B} = 0.01$ (and $c_{AB} = 0$). We defined $f = N_A/(N_A + N_B)$. We find that the stochastic decoupling noise induces an effective frequency-dependent fitness effect, which agrees well with the theoretical prediction, $c_{1B}(1 - f_0) - c_{1A}f_0 - c_{AB}(1 - 2f)$.

bioRxiv preprint doi: https://doi.org/10.1101/2024.02.23.581776; this version posted August 4, 2024. The copyright holder for this preprint (which was not certified by peer review) is the author/funder, who has granted bioRxiv a license to display the preprint in perpetuity. It is made available under aCC-BY 4.0 International license.

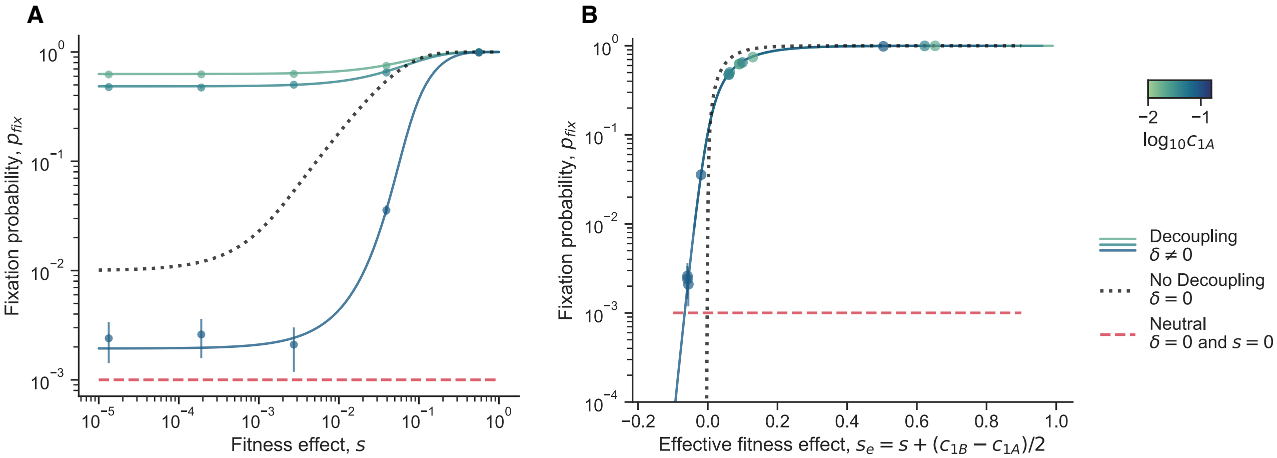

Figure S14: The dependence of p_{fix} on s_e (related to Figure 7). We show simulation results (round markers) and analytical predictions (solid lines) for the fixation probability, keeping δ constant but varying c_{1A}. We consistently use $\rho = 0$, so that $c_{1B} = \delta - c_{1A}$. We show the fixation probability as a function of (**A**) the raw fitness effect, s, and (**B**) the effective (constant) fitness effect $s_e = s + (c_{1B} - c_{1A})/2$. When we consider the fixation probability as a function of s_e, we see that the curves collapse into a single line. Additional parameter values used: $\delta = 0.2$, $f_0 = 10^{-2}$, and $N_e = 10^3$.

bioRxiv preprint doi: https://doi.org/10.1101/2024.02.23.581776; this version posted August 4, 2024. The copyright holder for this preprint (which was not certified by peer review) is the author/funder, who has granted bioRxiv a license to display the preprint in perpetuity. It is made available under aCC-BY 4.0 International license.

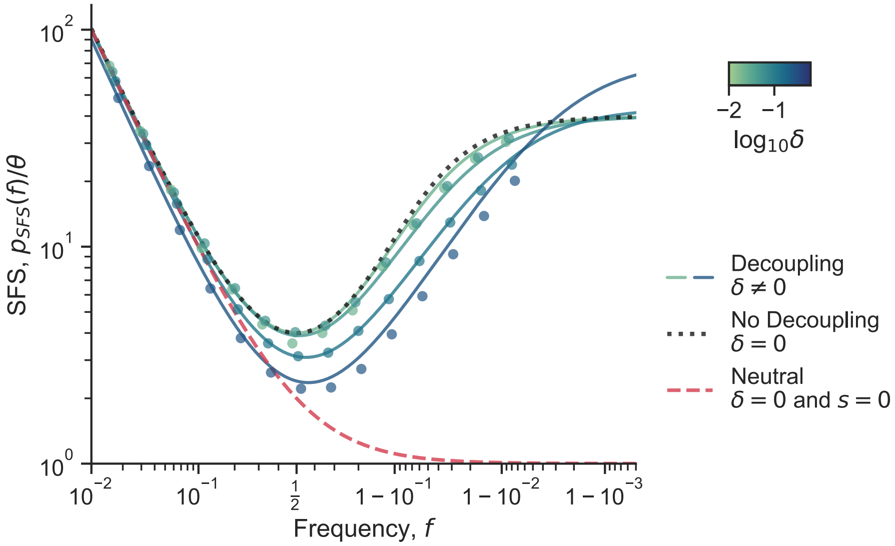

Figure S15: Site frequency spectrum with decoupling noise and constant selection. The same as Figure 7B, except where $s = 0.02$. The blue-green solid lines represent full analytical solutions for dynamics with decoupling noise, across different values of δ. The round markers show simulation results. The black dotted lines represent the case where there are no decoupling noise, but there is still (constant) natural selection. The red dashed lines represents the case where there is neither decoupling noise nor natural selection. We used $c_{1A} = c_{1B}$, $\rho_{AB} = 0.5$, $N_e = 10^3$ and $s = 0.02$.
